# Reduced Radial Curves of Diatomic Molecules

**DOI:** 10.1021/acs.jctc.3c00622

**Published:** 2023-09-29

**Authors:** Vladimír Špirko

**Affiliations:** Institute of Organic Chemistry and Biochemistry, p.r.i., Czech Academy of Sciences, Flemingovo nám. 2, 166 10 Prague 6, Czechia

## Abstract

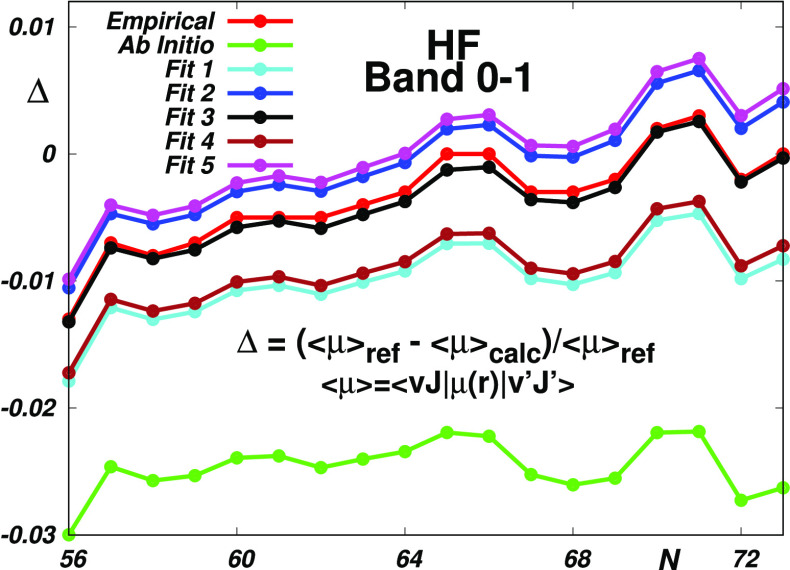

The prospect of using the concept of a universal reduced potential energy curve (RPC) for a broader class of radial molecular functions is explored by performing appropriate model calculations for the electric dipole moment functions of the hydrogen halides HF, HCl, and HBr. The reduced radial functions of the model systems, constructed from their best available theoretical approximants, coincide so closely that they can be used as few-parameter universal representations of functions available in the literature. Given the mathematical nature of the problem addressed here, the results are not limited to the functions studied but can be applied equally well to all radial molecular functions that have similar shapes, such as electric quadrupole moment and dipole polarizability functions.

## 1 Introduction

All fundamental concepts of molecular physics are based on the adiabatic approximation, in particular on the “zero-order” version of it introduced by Born and Oppenheimer, commonly referred to as the BO approximation.^1^ In this approximation, averaging over electronic degrees of freedom, one generates effective spin–rotation–vibration Hamiltonians involving a set of geometrically defined functions. For instance, for a ^1^Σ^+^ diatomic molecule in weak interaction with fixed external charges, one obtains:^2^
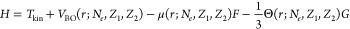
1where *T*_kin_ is the kinetic energy operator of the free molecule, *r* is the internuclear distance between two clamped nuclei, *V*_BO_(*r*) is the field-free BO potential energy curve, μ(*r*) and Θ(*r*) are the BO electric dipole and quadrupole moment curves, respectively, *Z*_1_ and *Z*_2_ are the atomic numbers of the two atoms, *N*_*e*_ is the number of molecular electrons, and *F* and *G* are the electric field and the field gradient due to the external fields, respectively. The same averaging can obviously be performed over any conceivable term (*H*_*X*_) of the general molecular Hamiltonian to obtain the corresponding “radial” function *X*(*r*; *N*_*e*_, *Z*_1_, *Z*_2_) and subsequently a purely “radial” spin–rotation–vibration Hamiltonian *H* for each electronic molecular state (see, e.g., refs (3,4)).

Formally, from a mathematical point of view, the radial functions of a diatomic system are based on the three fundamental parameters (*N*_*e*_, *Z*_1_, *Z*_2_) only. Unfortunately, however, there is no method in contemporary mathematics for explicitly constructing such functions.^5^ In practice, such functions can be evaluated straightforwardly using the first-principles quantum-chemical approaches (see, e.g., refs (6,7)) or, alternatively, by the Rydberg–Klein–Rees (RKR) inversion of experimental data (see, e.g., ref (8)). Still, both of these approaches suffer from serious drawbacks. Theoretical radial functions only rarely meet the accuracy required by spectroscopy, and the RKR inversion is severely limited by a notorious scarcity of inverted data and limited extrapolation beyond the classical turning points. Moreover, as first indicated by Trischka and Salwen^9^ in the case of μ(*r*) (see also ref (10)), an unambiguous determination of this function requires at least the knowledge of a complete row or column of the appropriate matrix <*v*|μ(*r*)|*v*′>.

To overcome these problems, it is increasingly common to apply a procedure in which the sought functions are determined by fitting their suitable mathematical approximants to available experimental data (see, e.g., refs (11−13)). Unfortunately, the choice of suitable approximants may be a complicated task because the shapes of the genuine functions are not known *a priori* and because the elementary mathematical functions available may not be flexible enough (see, e.g., refs (14−18) and references therein). Therefore, despite the broad variety of their literature variants (especially in the case of the potential energy function^19^), they easily become rather too awkward to allow “smooth” fitting. For instance, to describe quantitatively an *ab initio* potential energy function of He_2_ supporting only one bound state, Janzen and Aziz^20^ had to use 45 fitting parameters, and even the best approximants directly fitted to experimental data (see, e.g., ref (21)) may still not provide reliable predictions.

One of the ways of overcoming the above problems may consist of morphing approximate, but topologically correct, *ab initio* approximants by their fitting to accurate experimental data. Where potential energy functions of diatomic molecules are concerned, this procedure certainly appears to be a suitable tool when it is based on the RPC method of Jenč.^5,22,^ For instance, it has enabled the proper spectral assignment of highly excited vibrational states using the experimental *s*-wave scattering length^24^ and has helped to reveal the existence of the 12th vibrational state of the beryllium dimer and its two rotational states.^25^ The same approach can obviously be used for modeling any pair potential, including interaction potentials measured by atomic force spectroscopy;^26^ note that potentials modeled this way significantly deviate from those produced by the usually used Lennard–Jones model.^27^

The aim of this study is to adopt the RPC approach for molecular electric dipole moment (μ(*r*)), quadrupole moment (Θ(*r*)), and dipole polarizability (α_*zz*_(*r*)) functions. Like the potential energy function, μ(*r*), Θ(*r*), and α_*zz*_(*r*) are fundamental molecular characteristics (see, e.g., ref (28) and references therein) and have been studied both experimentally and theoretically in some detail (see, e.g., refs (29−36) and references therein). As accurate experimental data are available only for very low vibrational states, particularly for Θ(*r*) and α_*zz*_(*r*), most of these studies focus on the region around the equilibrium internuclear distance. To allow for the probing of higher-lying vibrational states, some such studies have attempted to construct appropriate dipole and quadrupole moment functions over a broad range of internuclear distances *r* by combining limited experimental data with reliable *ab initio* approximants. The resulting functions, obtained either as Padé approximants^32^ or as piecewise-continuous functions,^37−39^ exhibit physically correct shapes at small and large internuclear separations and closely coincide with genuine dipole and quadrupole moment and polarizability functions in the equilibrium region. However, although contemporary theory provides the sought-after radial functions quite accurately (see, e.g., refs (40−42)), it may still not provide results with experimentally achievable accuracy (see, e.g., refs (43−45)). Moreover, a close description of such calculated functions by means of Padé approximants or piecewise-continuous functions requires too many free parameters to enable their accurate fitting to experimental observations. Promisingly, as the probed electric functions topologically coincide with their potential energy counterparts (see below), it appears natural to assume that they could be morphed within the framework of Jen’s RPC “physics-guided” scheme using only a few fitting parameters. To verify this assumption, the author decided to perform relevant calculations for the hydrogen halides HF, HCl, and HBr, whose electric radial functions are among the best studied in the literature.

## 2 Theory

The reduced radial property curve (RRC) approach based on the RPC of Jen consists of two steps: First, a given single-minimum (reference) radial function *X*^ref^(*r*) (X = *V*, μ, Θ, α_*zz*_) is used to generate its reduced form *x*(ρ) (*x* = *u*, *m*, θ, *a*_*zz*_), which is defined as follows:
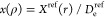
2where *D*_e_^ref^ is the depth of *X*^ref^(*r*) and the reduced variable ρ is related to *r* via the expression
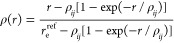
3Here, *r*_e_^ref^ is the distance for which *X*^ref^(*r*) acquires its minimum and ρ_*ij*_ satisfies the transcendental equation
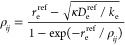
4where
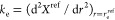
5and, finally, κ is a “universal-shape” constant (κ can be any constant allowing numerically stable solution of the transcendental equation, eq 4, for ρ_*ij*_).

In the second step, the reducing procedure is reverted by expressing *X*(*r*) as a function of *x*(ρ), namely
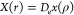
6with ρ defined by
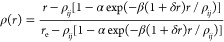
7and involving the *a priori* unknown parameters *D*_e_, *r*_e_, ρ_*ij*_, α, β, and δ, which are to be determined by fitting the experimental data available (in the standard RRC scheme, α = β = 1 and δ = 0).

Morphing of radial functions different from potential energy functions can be conveniently performed by fitting the “observed” matrix elements ⟨*vJ*|*X*(*r*)|*v*′*J*′⟩, which are usually represented as power series in the vibrational *v* and rotational *J* quantum numbers, respectively (see, e.g., ref (46)).

The ro-vibrational wave functions |*vJ* > are obtained by solving the Schrödinger equation for the following effective ro-vibrational Hamiltonian for an isolated ^1^Σ^+^ state^47^
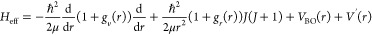
8where *V*_BO_ is the “mass-independent” part of the molecular potential energy curve (assumed to include the Born–Oppenheimer and relativistic terms) and the terms *V*′(*r*), *g*_*r*_(*r*), and *g*_*v*_(*r*) account for QED, residual retardation, adiabatic, and nonadiabatic effects. The sum *V*_eff_ = *V*_BO_(*r*) + *V*′(*r*) is assumed to be determinable by fitting to the experimental data available; relying on the results obtained in refs (4,48), the rotational *g*_*r*_(*r*) factor function is tentatively expressed as *g*_*r*_(*r*) = *g*_0_ + *g*_1_(*r* – *r*_*e*_)/(*r* + *r*_*e*_)^2^, where *g*_0_ and *g*_1_ are fitting parameters and the vibrational *g*-factor *g*_*v*_(*r*) is neglected.

## 3 Results and Discussion

**Figure 1:**
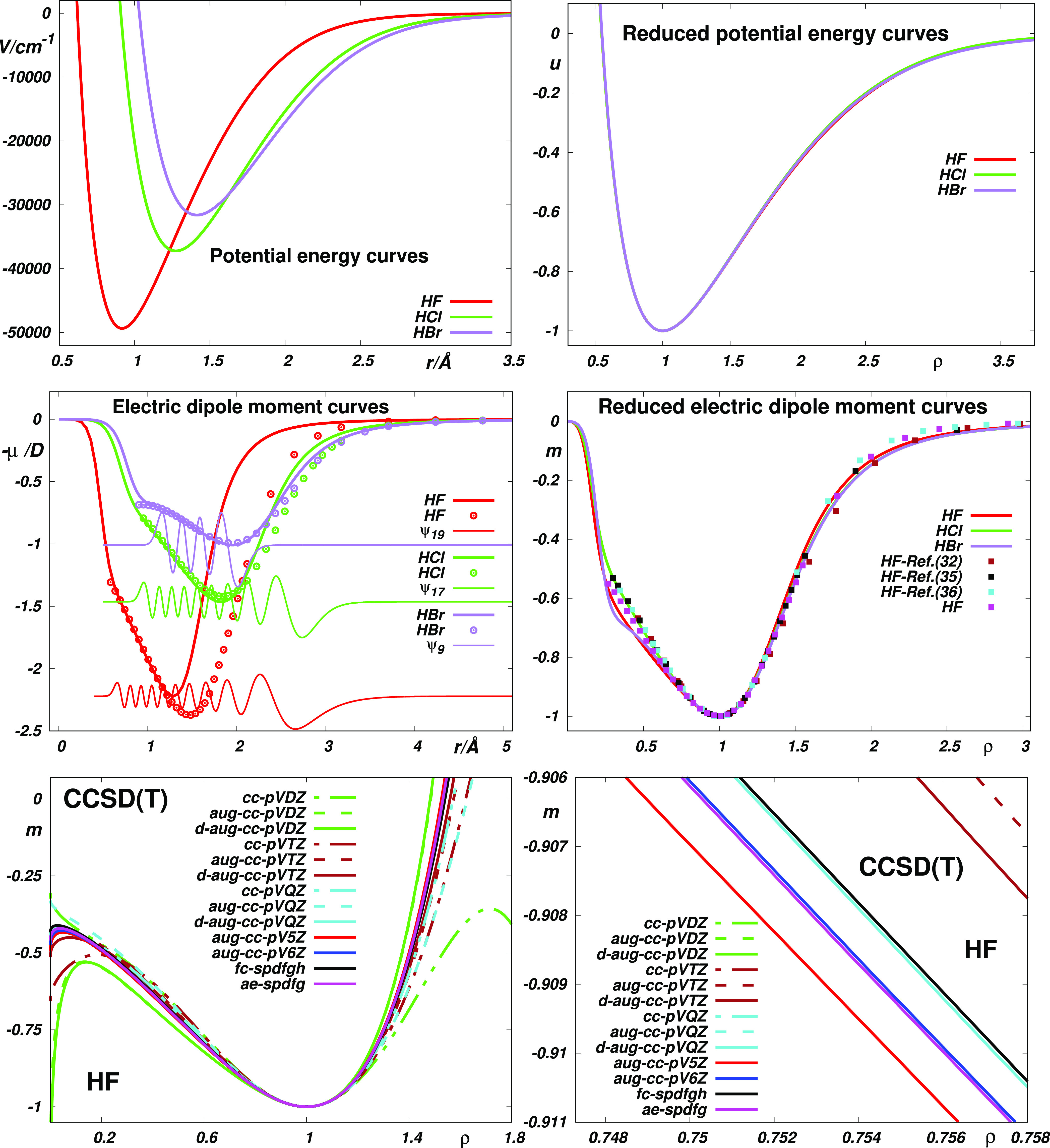
Potential and electric dipole moment functions of HF, HCl, and HBr and their reduced forms (top panels). The potential energy functions are taken from ref (33). The electric dipole and reduced electric dipole moment functions given in the middle panels are taken from ref (34) (solid lines) and from ref (31) (points), unless stated otherwise. The reduced electric dipole moment functions given in the bottom panels are constructed from the theoretical electric dipole moment functions given in ref (42).

To illustrate its properties, we first used the probed RRC scheme to construct the potential energy functions needed for generating the appropriate vibrational basis sets. As can be seen in the top panels of Figure 1 and deduced from Tables S1–S5, the scheme enables the construction of highly accurate RPC curves not only by morphing highly accurate empirical potential energy functions but also when using their less accurate *ab initio* approximants or even potential energy functions of chemically similar molecules while relaxing the “correction” parameters α, β, and δ (compare Fit 2 and Fit 4 of Table S1 and inspect Tables S4 and S5; it should be also said that the morphed RPC curves characterized in Tables S1–S5 coincide with their reference curves so closely that they cannot be distinguished grafically).

Interestingly, a similar situation also occurs in the case of electric dipole moments μ (see middle panels of Figure 1) and, to some extent, also in the case of electric quadrupole moments (Θ) and static dipole polarizabilities (α_*zz*_) (see Figure S1). However, because the level of accuracy achievable by both experiments and theory is only rarely comparable to that of data determining potential energy functions (see, e.g., ref (49)), the dispersion of reduced electric curves constructed from available literature data is greater than that of corresponding potential energy curves. Moreover, as seen, for example, in Figures S2 and S3, illustrating 40 theoretical EDM functions evaluated in ref (42) for HF and their reduced counterparts, most of the available “electric” functions possess unphysical asymptotes. Nonetheless, as can also be seen in the same figures, the curves available may acquire adequate shapes over the whole region of the vibrational displacements needed for a correct evaluation of the needed wave functions, and the slow convergence of the dispersion of the probed theoretical curves to their theoretical limit can be significantly enhanced by using explicitly correlated methods.^40−42^ Still, however, as shown in the bottom panels of Figure 1, differently augmented and correlated methods provide slightly different reduced curves, thus indicating a limited degree of their “universality” and the need to assess the role of this limit.

**Figure 2:**
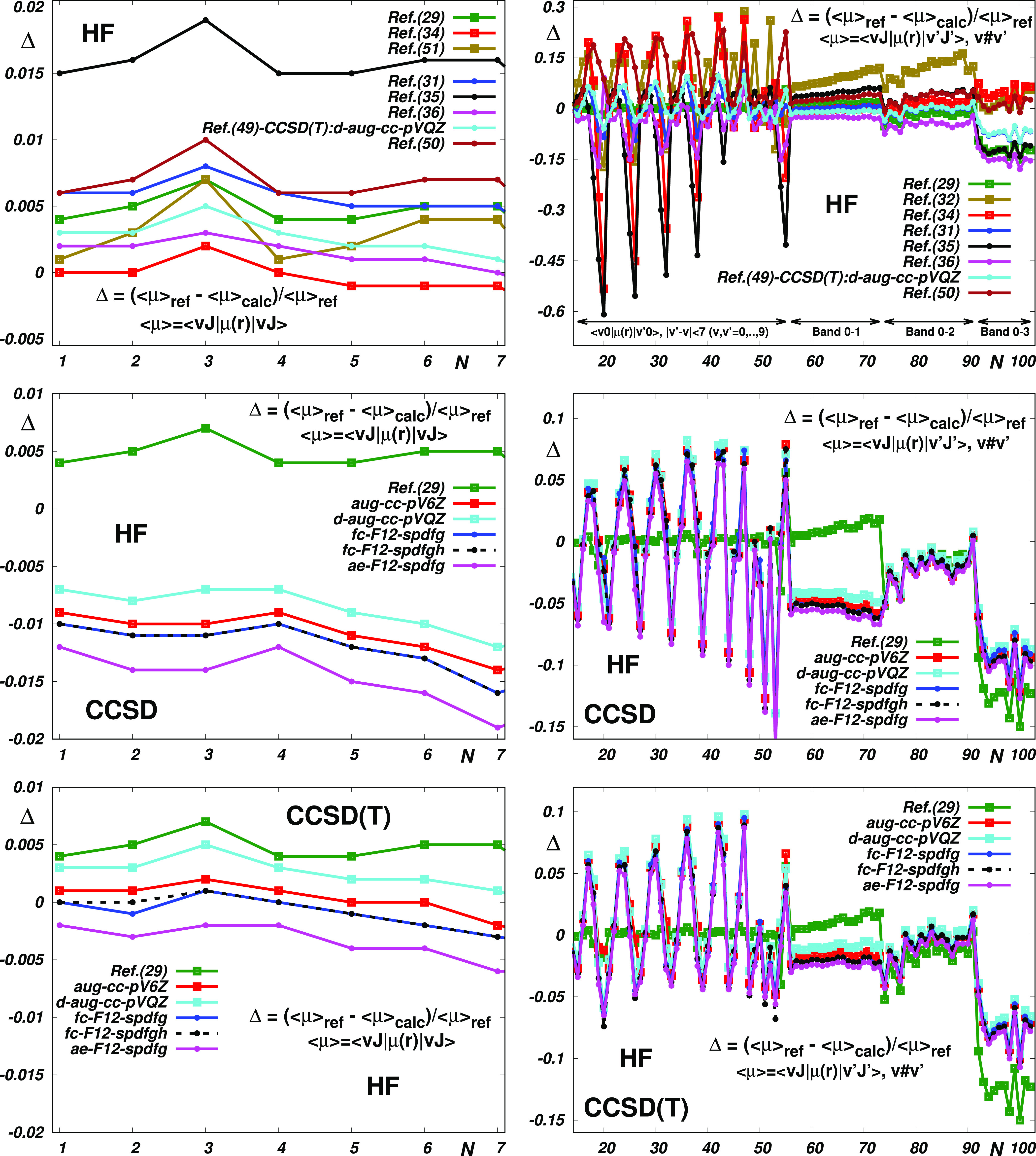
Reproduction of the HF reference data (see Table S6) by the “best” empirical^29,32,34^ and theoretical^31,35,36,50^ electric dipole moment functions (EDMs) and by their most accurate CCSD and CCSD(T) counterparts given in ref (42).

**Figure 3:**
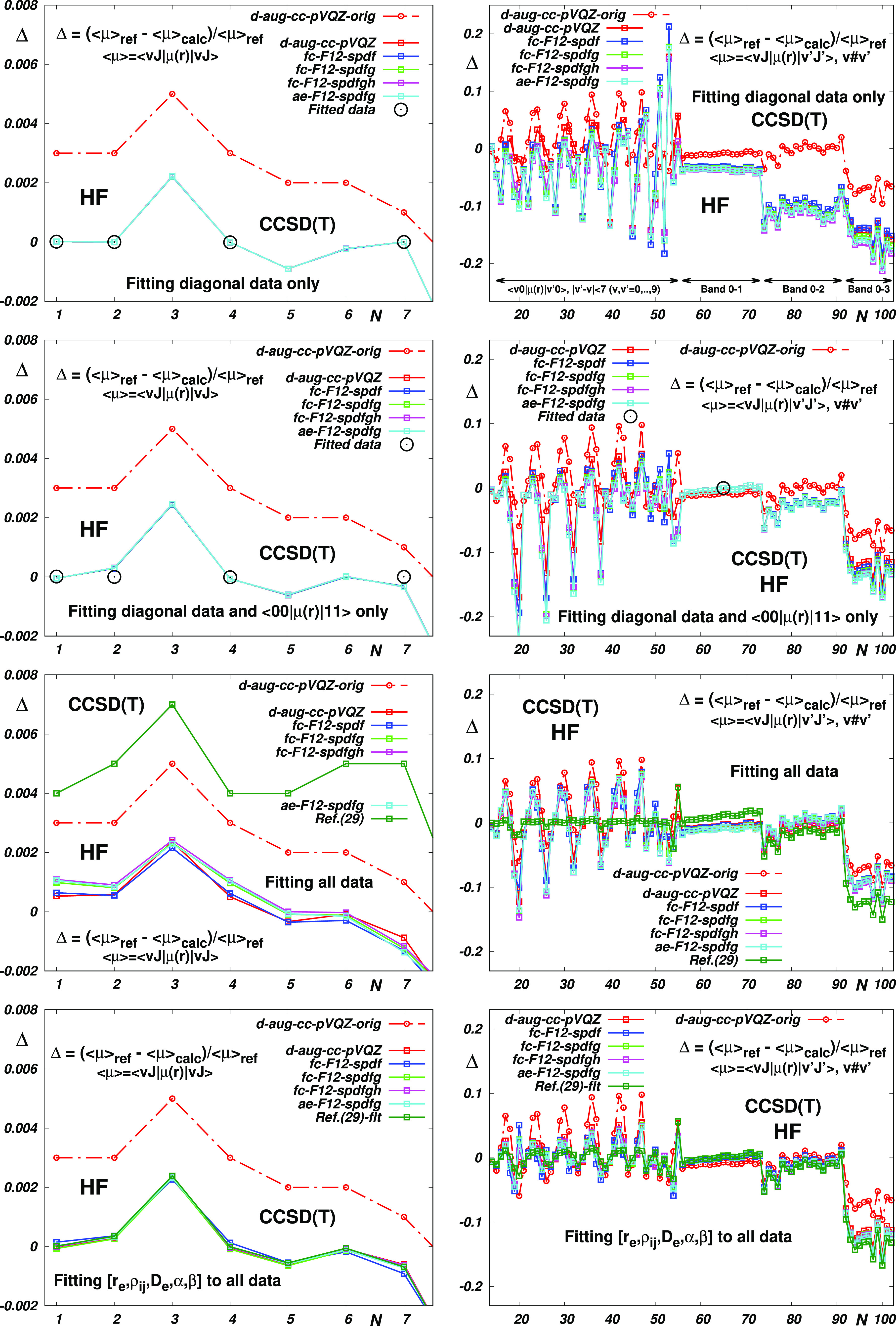
Reproduction of the HF reference data (see Table S6) by the electric dipole moment functions fitted using the reduced forms of the most accurate EDMs of ref (42) the original d-aug-cc-pVQZ CCSD(T) EDM function of ref (42) and the original empirical EDM function of ref (29) and its reduced version. Top panels: fitting the basic parameters (*r*_*e*_, ρ_*ij*_, *D*_*e*_) to <00|μ|00>, <10|μ|10>, <30|μ|30>, and <00|μ|11>. Bottom panels: fitting to all of the selected data using the basic (*r*_*e*_, ρ_*ij*_, *D*_*e*_) and extended (*r*_*e*_, ρ_*ij*_, *D*_*e*_, α, β) sets of the Jenč’s parameters, respectively.

Obviously, a simple visual comparison of the probe curves can be misleading. To provide better insight into their usage as fitting functions for physically correct interpolation and extrapolation, it is more appropriate to compare their ability to reproduce available data. Taking into account the amount of literature that has been published on relevant experimental measurements and theoretical calculations, it seems particularly appropriate and beneficial to perform these model calculations for the electrical dipole moment functions of the ground electronic states of hydrogen halides. To arrange for it, reference data sets have been generated mostly from the experimental data recommended in ref (34) and from the “best” available theoretical predictions, increasing the representativeness of these data sets (see Tables S6 and S8–S12). Subsequently, these data were compared to data calculated by using empirical and theoretical functions from the literature and their reduced counterparts constructed in this study. Regarding HF, in Figure 2, we can see that although the corresponding empirical and theoretical dispersion curves differ significantly, they still exhibit topologically very similar vibrational dependencies (two shapes are topologically equivalent if one can be transformed into the other without any cutting or gluing). As is seen in the bottom panels of Figure 2, a particularly close coincidence is exhibited by the curves obtained using explicitly correlated methods in ref (42), evidencing thus their suitability for the performed modeling. Expectably, as seen in Figure 3, the same dispersion is exhibited also by the curves obtained by “dereducing” the reduced forms of the original electric dipole moment functions. In Figure 3, one can also see that while the “best” empirical EDM function^29^ reproduces the reference off-diagonal <*v*|μ(*r*)|*v*′> data far better than its “best” theoretical counterparts, the theoretical functions are similarly better at describing the diagonal <*vJ*|μ(*r*)|*vJ*> data. Interestingly (see the bottom panels), when morphing the “best” empirical EDM of (29) on the one hand and using the correction parameters α and β for the theoretical EDMs on the other, one obtains fairly comparable results for both types of EDM curves used. It should be noted, however, that the data of ref (29) have large reported uncertainties (∼15%) and that the fitting accuracy of the latter results is thus “numerical” rather than “physical”. In any case, the results obtained using the theoretical EDMs seem to enable the reproduction of the observed data within their error bars using only three basic parameters (see Table S7), whereas “accurate” empirical EDM functions usually require the inclusion of more than twice as many fitting parameters.

**Figure 4:**
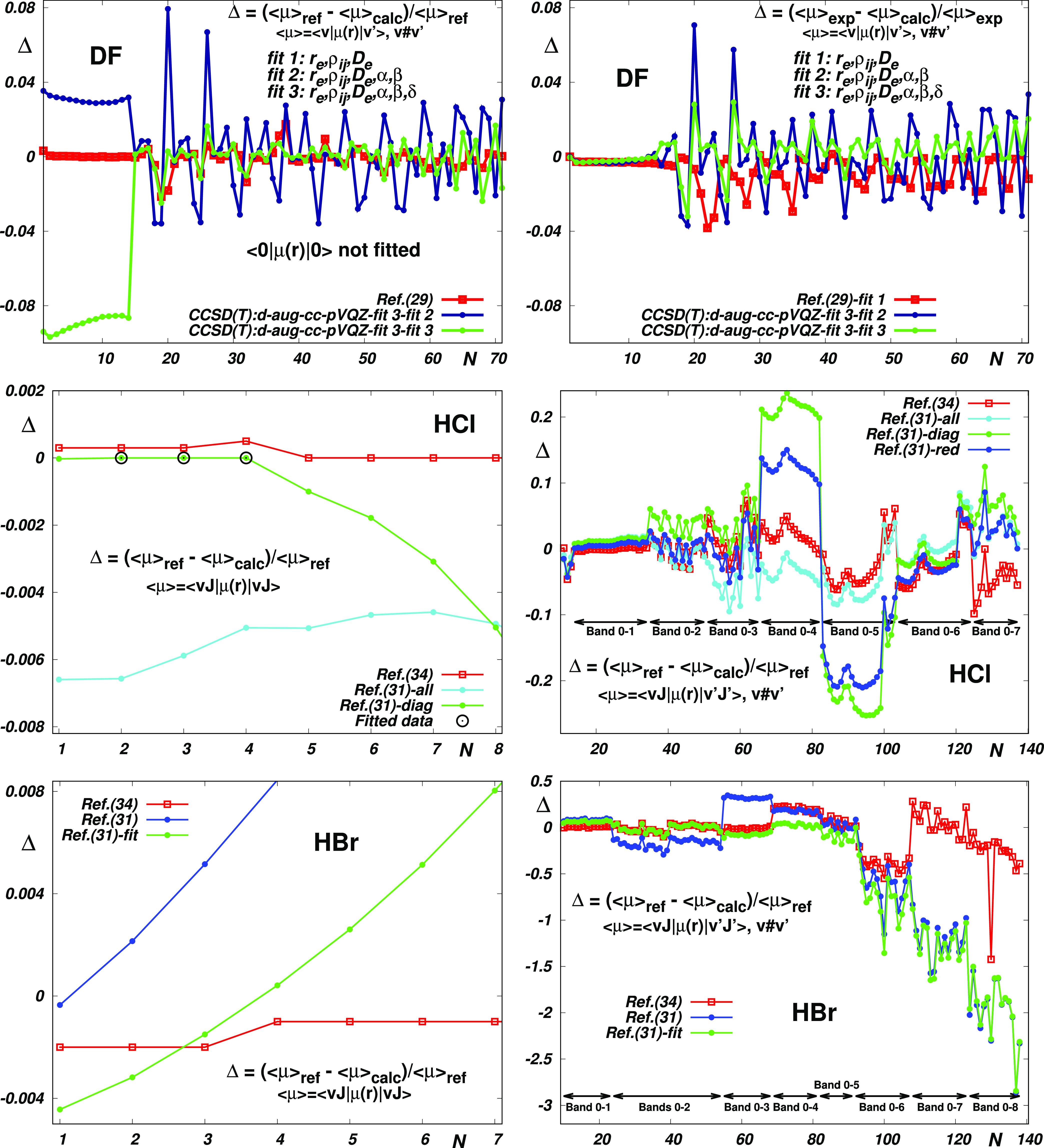
Reproduction of the DF, HCl, and HBr reference data (see Tables S8–S12) by the empirical and theoretical electric dipole moment functions and by their morphed variants obtained by fitting the reference data. Unless stated otherwise, the morphing was performed using the “basic” parameters *r*_*e*_, ρ_*ij*_, and *D*_*e*_. The ref(31)-all and ref(31)-diag results presented for HCl (middle panels) were obtained by fitting all respected and only “diagonal” data, respectively. The ref(31)-fit results presented for HBr were obtained by fitting to the *v* < 6 data only.

As concerns the remaining probed models (see Figure 4 and Tables S8–S12), one can arrive at similar conclusions as in the case of HF. The following two facts are worth mentioning explicitly: (a) Morphing the “best” CCSD(T) reduced EDM functions of ref (42) while respecting the correction parameters α, β, and δ provides EDM functions reproducing available experimental data of DF equally well as the “best” (many-parameter) empirical EDM of ref (29). (b) Morphing the best available (MRCI) reduced EDM functions of HCl and HBr^31^ by fitting to the best available experimental data reveals profound incompatibility of the experimental matrix elements <0|μ(*r*)|4> and <0|μ(*r*)|5> of HCl and (<0|μ(*r*)|6>, <0|μ(*r*)|7> and <0|μ(*r*)|8>) of HBr with the rest of available data, questioning thus the physical adequacy of corresponding empirical functions from the literature. Interestingly, in full agreement with the results, a very recent experimental study^51^ on H^35^Cl and H^37^Cl reports line intensities for the 5–0 bands, which are about 23% greater than the reference values of ref (34)

## 4 Conclusions

The concept of a universal reduced radial curve (RRC) allowing the systematic study of radial functions of diatomic molecules in a unified scheme has been probed by performing actual numerical calculations for ground electronic states of the halide hydrides HF(DF), HCl, and HBr. Within the framework of this scheme, the radial functions of different molecules and different molecular states, both empirical and theoretical, may be directly compared, and their regularities/irregularities may be conveniently visualized. Being “physics-guided”, the RRC scheme enables the construction of reliable radial functions over a large range of interatomic distances using a much smaller number of fitting parameters than the number of fitting parameters needed when using usual polynomials or splines. The approach appears to be especially advantageous for “noisy” transition intensity data exhibiting high uncertainties, which cannot be fitted safely using low-order polynomials.

## Supporting Information Available

Reduced potential energy and electric dipole moment curves and reference values of ro-vibrational electric dipole matrix elements of hydrogen halides (PDF)

## Dedication

This paper is dedicated to the memory of Prof. Josef Paldus.
