# Supplementary material for: Reduced Radial Curves of Diatomic Molecules
Source: J Chem Theory Comput. 2023 Sep 29;19(20):7324–32. doi: 10.1021/acs.jctc.3c00622 (PMC10601484; doi:10.1021/acs.jctc.3c00622)
Supplement: Supplementary file 1 — ct3c00622_si_001.pdf [file ct3c00622_si_001.pdf]

Supporting Information. Reduced Radial Curves of Diatomic Molecules.

Vladimír Špirko *

Institute of Organic Chemistry and Biochemistry, p.r.i., Czech Academy of Sciences, Flemingovo nám. 2, 166 10 Prague 6, Czechia

E-mail: spirko@uochb.cas.cz

Phone: +420 220183 571

Table S1: RPC parameters of HF and DF in the $X^1\Sigma^+$ state determined by morphing the potential energy curves of Refs.[52,53] by a separate fitting to experimental ro-vibrational spectroscopic constants taken from Ref.[52 a].

Parameter	HF	HF	HF	HF	DF	DF
Fit No.	1	2	3	4	5	6
r_e, Å	0.916851(1)	0.9168320(3)	0.9168429(5)	0.9168351(7)	0.9169111(1)	0.9169166(4)
ρ_{ij}, Å	0.296239(5)	0.296175(1)	0.295004(2)	0.29519(5)	0.296314(1)	0.296338(4)
D_e, cm^{-1}	49355.9(6)	49360.3(1)	49510.9(2)	49504(2)	49350.6(2)	49348.6(5)
g_o	0.0	0.0	0.0	0.0	0.0	0.0
g_1	0.0	*-0.0068*	*-0.0075*	*-0.0068*	*-0.0039*	0.0
α	1.0	1.0	1.0	1.0357(50)	1.0	1.0
β	1.0	1.0	1.0	1.0299(42)	1.0	1.0
δ	0.0	0.0	0.0	-0.0223(34)	0.0	0.0
σ^b_{fit}, cm^{-1}	0.188	0.050	0.080	0.045	0.009	0.028

[a]Unless stated otherwise, morphing was performed using the reference (HF) potential energy curve of Ref.[52]; Fit 3 and Fit 4 were performed using the nonrelativistic potential of Ref.[53]; the values given in italics were fixed after a preliminary determination. [b]The standard deviation of the fit.

Table S2: RPC parameters of HCl and DCl in the $X^1\Sigma^+$ state (Fits 1–4) determined by morphing the Born–Oppenheimer potential energy curve of Ref.[54] by a separate fitting to experimental ro-vibrational spectroscopic constants taken from[54 a].

Parameter	$H^{35}Cl$	$H^{37}Cl$	$D^{35}Cl$	$D^{37}Cl$	$H^{35}Cl$
Fit No.	1	2	3	4	5
r_e, Å	1.274171(22)	1.274168(22)	1.274040(10)	1.274045(11)	1.276021(6)
ρ_{ij}, Å	0.589034(31)	0.589028(30)	0.589209(14)	0.589200(15)	0.63727(48)
D_e, cm^{-1}	37229.7(13)	37229.9(13)	37214.1(5)	37214.4(5)	36703(13)
g_o	-0.000582(34)	-0.000582(33)	-0.000845(16)	-0.000837(16)	*0.0022*
g_1	*-0.009*	*-0.009*	*-0.002*	*-0.002*	0.0
α	1.0	1.0	1.0	1.0	0.78733(73)
β	1.0	1.0	1.0	1.0	0.7623(11)
δ	-0.00149(11)	-0.00148(10)	-0.00184(4)	-0.00181(4)	0.0
σ^b_{fit}, cm^{-1}	0.021	0.020	0.009	0.009	0.188

[a]Fit 5 performed using the HF nonrelativistic potential of Ref.[53]; the values given in italics were fixed after a preliminary determination. [b]The standard deviation of the fit.

Table S3: RPC parameters of HBr and DBr in the $X^1\Sigma^+$ state (Fits 1–4) determined by morphing the Born–Oppenheimer potential energy curve of Ref.[55] by a separate fitting to experimental ro-vibrational spectroscopic constants taken from Ref.[55a].

Parameter	$H^{79}Br$	$H^{81}Br$	$D^{79}Br$	$D^{81}Br$	$H^{79}Br$
Fit No.	1	2	3	4	5
r_e, Å	1.413853(36)	1.413857(35)	1.414070(20)	1.414070(20)	1.416135(7)
ρ_{ij}, Å	0.751459(68)	0.751459(68)	0.751005(52)	0.751006(52)	0.83016(62)
D_e, cm^{-1}	31611.1(18)	31611.2(18)	31620.8(10)	31620.8(10)	30652(11)
g_o	-0.000795(50)	-0.000789(49)	-0.000547(27)	-0.000547(27)	*0.0023*
g_1	*-0.015*	*-0.015*	*-0.003*	*-0.003*	0.0
α	1.0	1.0	1.0	1.0	0.79425(52)
β	1.0	1.0	1.0	1.0	0.76320(92)
δ	-0.00106(12)	-0.00105(12)	0.0	0.0	0.0
σ^b_{fit}, cm^{-1}	0.029	0.029	0.008	0.008	0.173

[a]Fit 5 performed using the HF nonrelativistic potential of Ref.[53]; the values given in italics were fixed after a preliminary determination. [b]The standard deviation of the fit.

Table S4: Vibrational energies E^v(in cm^{-1}) of the $X^1\Sigma^+$ state of HF, HCl, and HBr[a].

v	E^v_{calc}	$E^v_{ref}-E^v_{calc}$	weight	E^v_{calc}	$E^v_{ref}-E^v_{calc}$	weight	E^v_{calc}	$E^v_{ref}-E^v_{calc}$	weight
		HF			$H^{35}Cl$			$H^{79}Br$	
0	[2050.7621]	[-0.0065]		[1483.8044]	[0.0762]		[1314.6149]	[0.0377]	
1	3961.4224	0.0047	50	2885.9822	-0.0059	50	2558.9511	-0.0376	50
2	7750.7934	0.0002	50	5668.0191	-0.0361	50	5027.3857	-0.0481	50
3	11372.8040	-0.0162	50	8346.8212	-0.0443	50	7405.2846	-0.0238	50
4	14831.6421	0.0020	50	10922.8149	0.0142	50	9692.3210	0.0381	50
5	18130.9394	0.0047	50	13396.2143	0.0608	50	11887.8702	0.0623	50
6	21273.7138	0.0312	10	15766.8019	0.0641	50	13990.7980	0.0208	50
7	24262.3012	0.1015	10	18033.9590	-0.0621	50	15999.4090	-0.0872	50
8	27098.0734	0.1045	10	20196.4574	-0.3345	0	17911.1976	-0.0377	0
9	29781.5124	0.0336	10	22252.3402	-0.7039	0	19722.6026	-0.0377	0

[a]E^v_{ref} taken from Ref.[33], E^v_{calc} of HF, HCl and HBr calculated using Fit 4 of Table S1, Fit 5 of Table S2 and Fit 5 of Table S3, respectively.

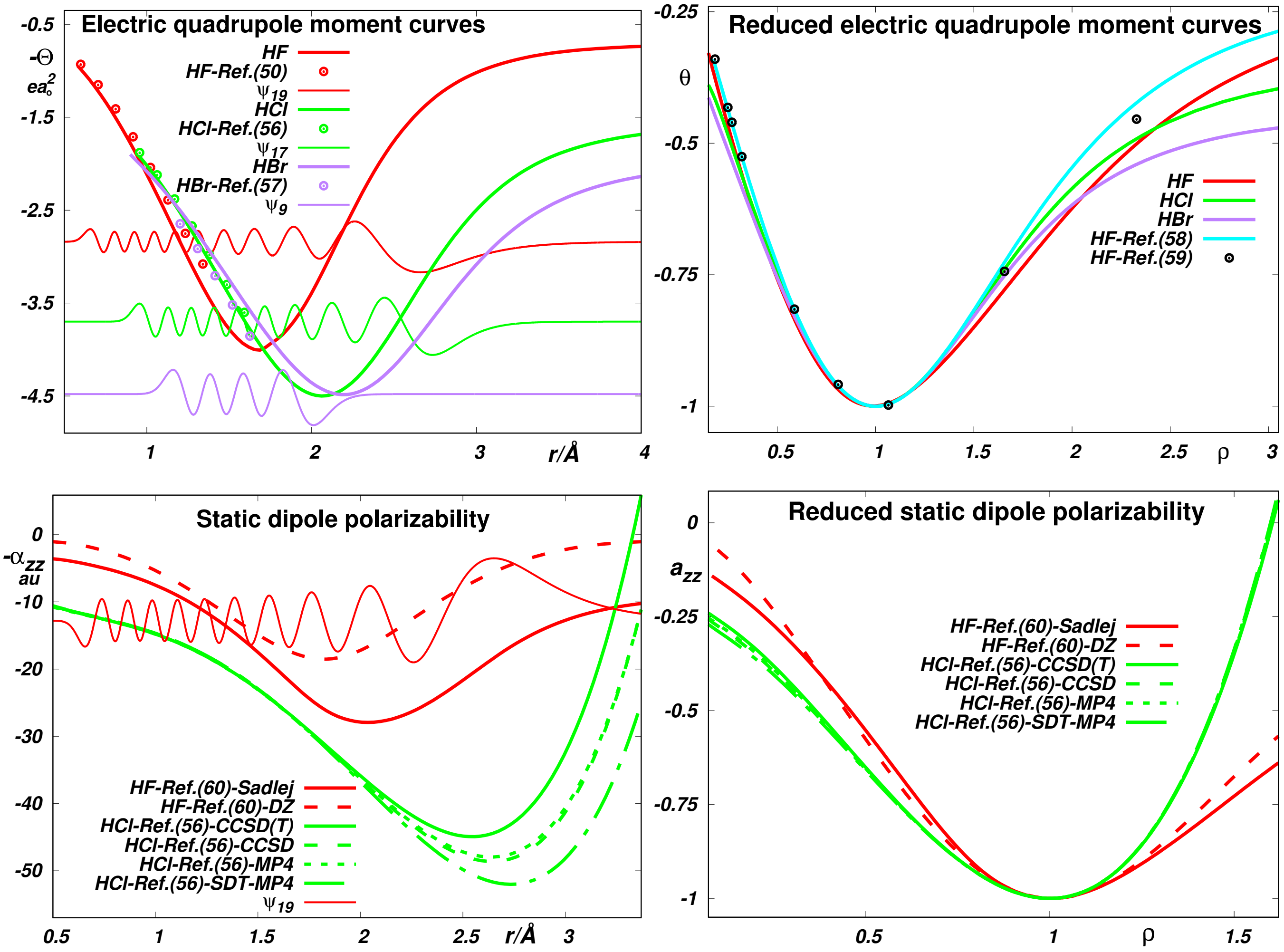

Fig. S1. Top panels: Electric quadrupole moment and reduced electric quadrupole moment functions of HF,HCl and HBr. Unless stated otherwise, the electric quadrupole moment functions are taken from Ref. [31]. Bottom panels: Dipole polarizability and reduced dipole polarizability functions of HF (Ref. [60]) and HCl (Ref. [56]). The vibrational wavefunctions plotted in the panels pertain to the highest states measured.

Table S5: Rotational energies E^r(in cm^{-1}) of the $X^1\Sigma^+$ state of HF^a.

v"	J"	v'	J'	E^r_{ref}	E^r_{calc}	$E^r_{ref} - E^r_{calc}$	weight	v"	J"	v'	J'	E^r_{ref}	E^r_{calc}	$E^r_{ref} - E^r_{calc}$	weight
0	0	0	1	41.1110	41.1053	0.0057	100.0	5	0	5	1	33.7501	33.7442	0.0059	100.0
0	0	0	2	123.2821	123.2780	0.0041	100.0	5	0	5	2	101.2054	101.2008	0.0046	100.0
0	0	0	3	246.4118	246.4098	0.0020	100.0	5	0	5	3	202.2763	202.2734	0.0029	100.0
0	0	0	4	410.3479	410.3484	-0.0004	100.0	5	0	5	4	336.8288	336.8279	0.0009	100.0
0	0	0	5	614.8884	614.8911	-0.0028	100.0	5	0	5	5	504.6844	504.6854	-0.0010	100.0
0	0	0	6	859.7812	859.7857	-0.0046	100.0	5	0	5	6	705.6210	705.6234	-0.0025	100.0
0	0	0	7	1144.7253	1144.7306	-0.0053	100.0	5	0	5	7	939.3728	939.3759	-0.0031	100.0
0	0	0	8	1469.3714	1469.3758	-0.0044	100.0	5	0	5	8	1205.6314	1205.6339	-0.0024	100.0
0	0	0	9	1833.3224	1833.3235	-0.0011	100.0	5	0	5	9	1504.0462	1504.0461	0.0001	100.0
0	0	0	10	2236.1346	2236.1292	0.0053	100.0	5	0	5	10	1834.2248	1834.2199	0.0050	100.0
0	0	0	11	2677.3183	2677.3027	0.0156	0.0	5	0	5	11	2195.7347	2195.7218	0.0129	0.0
0	0	0	12	3156.3393	3156.3087	0.0306	0.0	5	0	5	12	2588.1031	2588.0786	0.0245	0.0
0	0	0	13	3672.6200	3672.5687	0.0513	0.0	5	0	5	13	3010.8185	3010.7781	0.0404	0.0
0	0	0	14	4225.5403	4225.4618	0.0785	0.0	5	0	5	14	3463.3316	3463.2703	0.0613	0.0
1	0	1	1	39.5667	39.5609	0.0058	100.0	6	0	6	1	32.3662	32.3604	0.0058	100.0
1	0	1	2	118.6506	118.6462	0.0044	100.0	6	0	6	2	97.0547	97.0504	0.0043	100.0
1	0	1	3	237.1528	237.1503	0.0025	100.0	6	0	6	3	193.9778	193.9755	0.0023	100.0
1	0	1	4	394.9253	394.9249	0.0004	100.0	6	0	6	4	323.0041	323.0041	-0.0001	100.0
1	0	1	5	591.7712	591.7727	-0.0015	100.0	6	0	6	5	483.9587	483.9613	-0.0026	100.0
1	0	1	6	827.4452	827.4482	-0.0029	100.0	6	0	6	6	676.6240	676.6289	-0.0049	100.0
1	0	1	7	1101.6544	1101.6577	-0.0033	100.0	6	0	6	7	900.7397	900.7463	-0.0066	100.0
1	0	1	8	1414.0586	1414.0605	-0.0020	100.0	6	0	6	8	1156.0032	1156.0106	-0.0074	100.0
1	0	1	9	1764.2712	1764.2695	0.0016	100.0	6	0	6	9	1442.0708	1442.0774	-0.0066	100.0
1	0	1	10	2151.8601	2151.8518	0.0082	100.0	6	0	6	10	1758.5580	1758.5618	-0.0038	100.0
1	0	1	11	2576.3487	2576.3301	0.0186	0.0	6	0	6	11	2105.0402	2105.0385	0.0017	0.0
1	0	1	12	3037.2168	3037.1834	0.0335	0.0	6	0	6	12	2481.0535	2481.0430	0.0105	0.0
1	0	1	13	3533.9021	3533.8483	0.0537	0.0	6	0	6	13	2886.0958	2886.0725	0.0232	0.0
1	0	1	14	4065.8006	4065.7203	0.0803	0.0	6	0	6	14	3319.6275	3319.5869	0.0407	0.0
2	0	2	1	38.0619	38.0561	0.0058	100.0	7	0	7	1	31.0002	30.9942	0.0060	100.0
2	0	2	2	114.1374	114.1331	0.0043	100.0	7	0	7	2	92.9574	92.9525	0.0049	100.0
2	0	2	3	228.1304	228.1280	0.0024	100.0	7	0	7	3	185.7855	185.7821	0.0034	100.0
2	0	2	4	379.8964	379.8963	0.0002	100.0	7	0	7	4	309.3554	309.3537	0.0016	100.0
2	0	2	5	569.2439	569.2459	-0.0020	100.0	7	0	7	5	463.4953	463.4955	-0.0002	100.0
2	0	2	6	795.9339	795.9375	-0.0037	100.0	7	0	7	6	647.9912	647.9931	-0.0019	100.0
2	0	2	7	1059.6809	1059.6853	-0.0044	100.0	7	0	7	7	862.5873	862.5905	-0.0032	100.0
2	0	2	8	1360.1536	1360.1572	-0.0036	100.0	7	0	7	8	1106.9863	1106.9899	-0.0037	100.0
2	0	2	9	1696.9754	1696.9762	-0.0007	100.0	7	0	7	9	1380.8499	1380.8528	-0.0030	100.0
2	0	2	10	2069.7254	2069.7205	0.0049	100.0	7	0	7	10	1683.7997	1683.8004	-0.0007	100.0
2	0	2	11	2477.9391	2477.9251	0.0140	0.0	7	0	7	11	2015.4180	2015.4143	0.0036	0.0
2	0	2	12	2921.1098	2921.0824	0.0274	0.0	7	0	7	12	2375.2477	2375.2372	0.0106	0.0
2	0	2	13	3398.6894	3398.6435	0.0459	0.0	7	0	7	13	2762.7943	2762.7737	0.0206	0.0
2	0	2	14	3910.0894	3910.0192	0.0702	0.0	7	0	7	14	3177.5256	3177.4912	0.0343	0.0
3	0	3	1	36.5934	36.5875	0.0059	100.0	8	0	8	1	29.6450	29.6389	0.0061	100.0
3	0	3	2	109.7331	109.7285	0.0046	100.0	8	0	8	2	88.8926	88.8872	0.0054	100.0
3	0	3	3	219.3253	219.3223	0.0030	100.0	8	0	8	3	177.6576	177.6533	0.0043	100.0
3	0	3	4	365.2294	365.2283	0.0012	100.0	8	0	8	4	295.8129	295.8099	0.0030	100.0
3	0	3	5	547.2585	547.2590	-0.0005	100.0	8	0	8	5	443.1890	443.1874	0.0016	100.0
3	0	3	6	765.1796	765.1812	-0.0015	100.0	8	0	8	6	619.5748	619.5745	0.0003	100.0
3	0	3	7	1018.7145	1018.7161	-0.0016	100.0	8	0	8	7	824.7178	824.7185	-0.0007	100.0
3	0	3	8	1307.5401	1307.5401	-0.0000	100.0	8	0	8	8	1058.3244	1058.3256	-0.0012	100.0
3	0	3	9	1631.2891	1631.2853	0.0037	100.0	8	0	8	9	1320.0606	1320.0613	-0.0007	100.0
3	0	3	10	1989.5510	1989.5407	0.0103	100.0	8	0	8	10	1609.5525	1609.5516	0.0009	100.0
3	0	3	11	2381.8729	2381.8526	0.0203	0.0	8	0	8	11	1926.3868	1926.3825	0.0043	0.0
3	0	3	12	2807.7606	2807.7259	0.0347	0.0	8	0	8	12	2270.1115	2270.1019	0.0096	0.0
3	0	3	13	3266.6793	3266.6252	0.0540	0.0	8	0	8	13	2640.2367	2640.2191	0.0175	0.0
2	0	3	14	3758.0549	3757.9757	0.0792	0.0	8	0	8	14	3036.2349	3036.2065	0.0284	0.0
4	0	4	1	35.1575	35.1517	0.0058	100.0	9	0	9	1	28.2923	28.2858	0.0065	100.0
4	0	4	2	105.4266	105.4222	0.0044	100.0	9	0	9	2	84.8348	84.8284	0.0064	100.0
4	0	4	3	210.7157	210.7132	0.0024	100.0	9	0	9	3	169.5433	169.5370	0.0062	100.0
4	0	4	4	350.8876	350.8873	0.0002	100.0	9	0	9	4	282.2915	282.2856	0.0060	100.0
4	0	4	5	525.7598	525.7618	-0.0020	100.0	9	0	9	5	422.9117	422.9061	0.0056	100.0
4	0	4	6	735.1050	735.1088	-0.0038	100.0	9	0	9	6	591.1944	591.1892	0.0052	100.0
4	0	4	7	978.6515	978.6564	-0.0049	100.0	9	0	9	7	786.8890	786.8843	0.0047	100.0
4	0	4	8	1256.0839	1256.0885	-0.0046	100.0	9	0	9	8	1009.7042	1009.7000	0.0042	100.0
4	0	4	9	1567.0437	1567.0462	-0.0025	100.0	9	0	9	9	1259.3083	1259.3044	0.0038	100.0
4	0	4	10	1911.1300	1911.1279	0.0021	100.0	9	0	9	10	1535.3295	1535.3256	0.0039	100.0
4	0	4	11	2287.9004	2287.8907	0.0097	0.0	9	0	9	11	1837.3568	1837.3523	0.0045	0.0
4	0	4	12	2696.8722	2696.8509	0.0213	0.0	9	0	9	12	2164.9401	2164.9342	0.0059	0.0
4	0	4	13	3137.5228	3137.4855	0.0373	0.0	9	0	9	13	2517.5911	2517.5825	0.0085	0.0
4	0	4	14	3609.2914	3609.2326	0.0588	0.0	9	0	9	14	2894.7834	2894.7708	0.0126	0.0

[a] E^r_{ref} taken from Ref.[52], E^r_{calc} calculated using the (Fit 4,Table S1) potential energy function obtained by morphing its nonrelativistic *ab initio* approximant taken from Ref.[53].

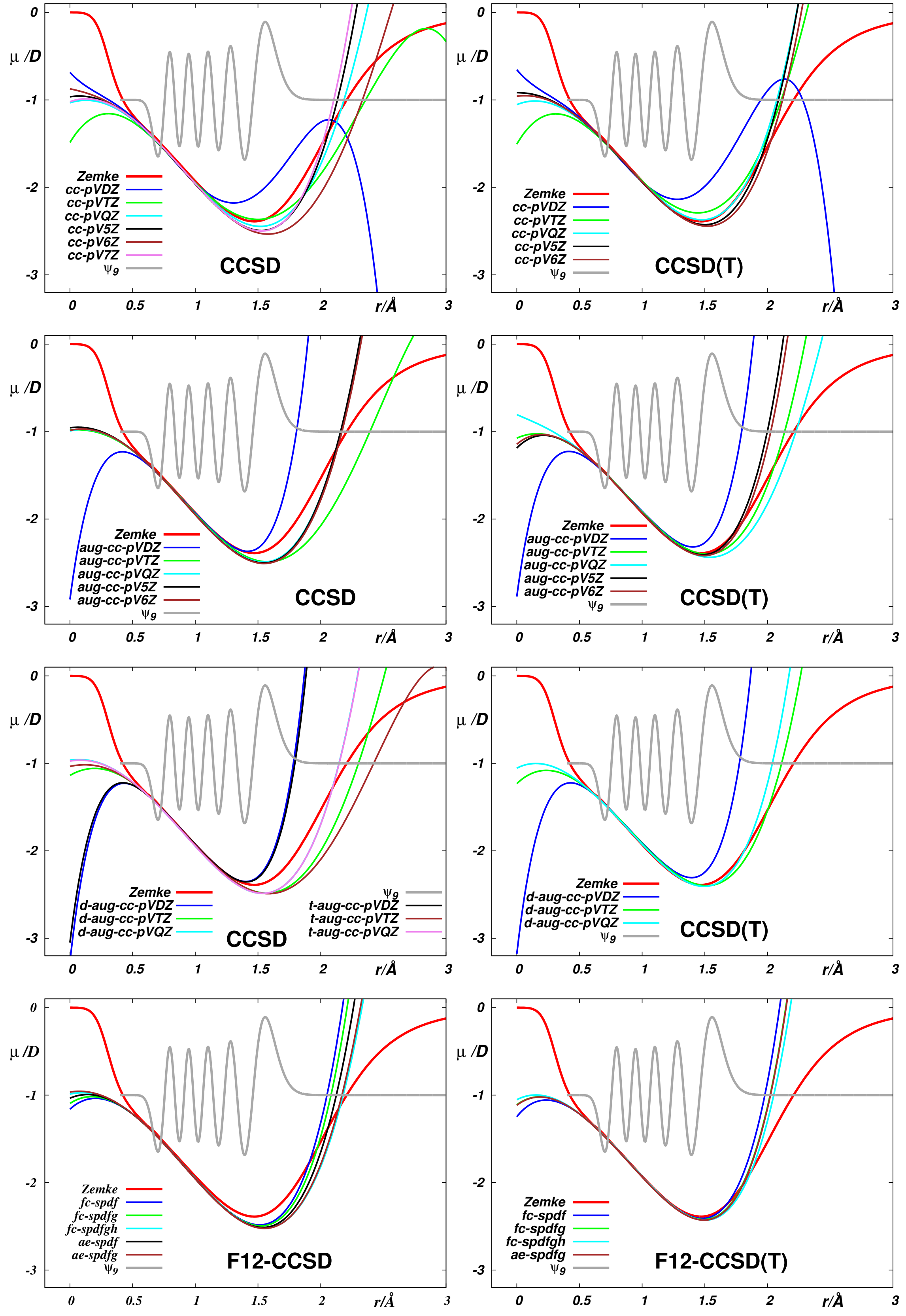

Fig. S2. Comparison of the polynomial dipole moment functions of HF evaluated in Ref.[42] (see Table S2 of Ref.[42]) with their analogue obtained by amending the function that *Zemke* evaluated in Ref.[36] by the $r \to 0$ asymptote evaluated in Ref.[38]. ψ_9 is the wavefunction of the so far highest experimentally probed state v=9 (Ref.[29]).

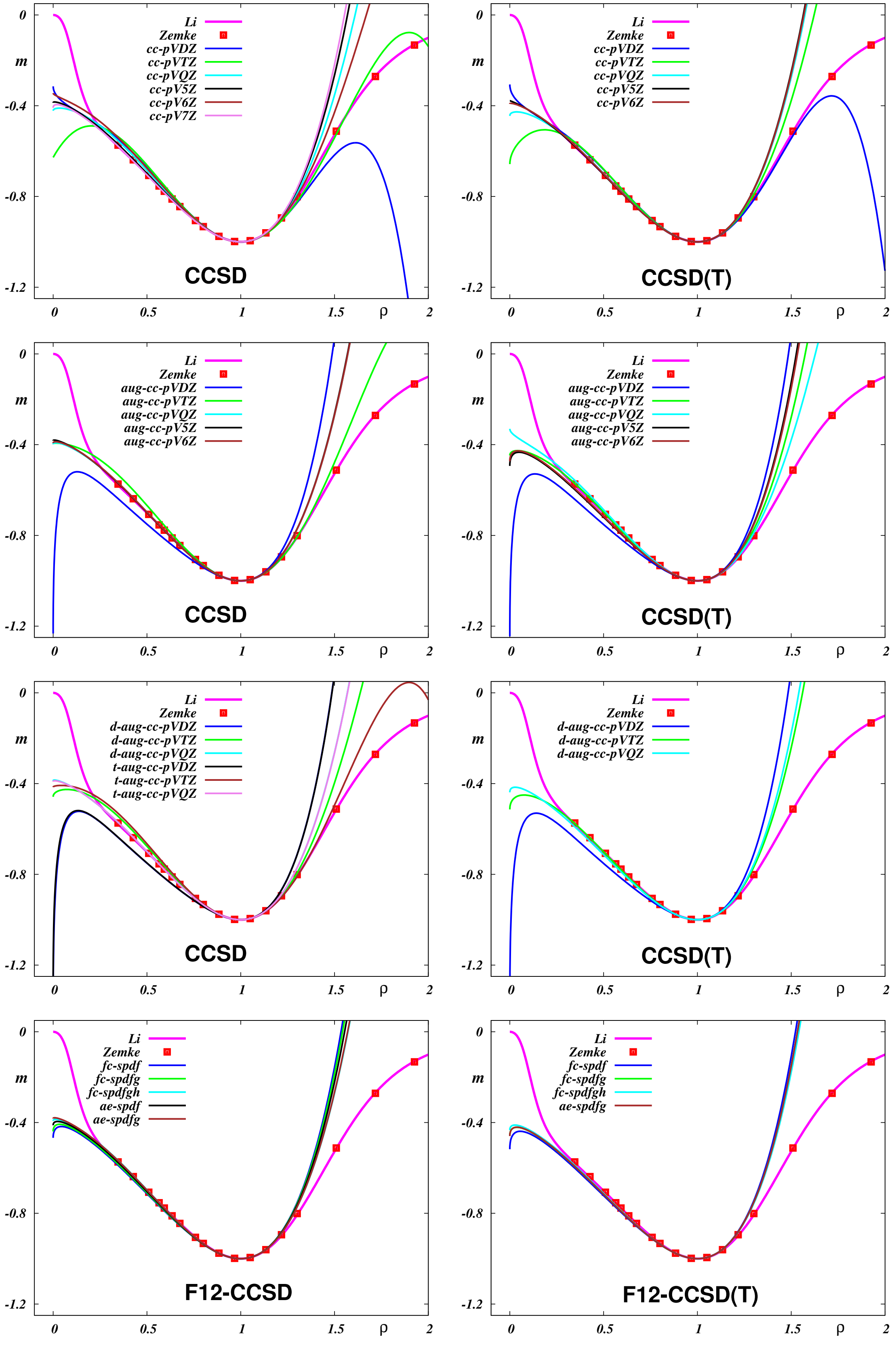

Fig. S3. Comparison of the reduced dipole moment functions of HF pertaining to the dipole moment functions plotted in Fig. S1 with their analogues obtained by reducing the empirical dipole moment *Li* of Ref.[34] and the *ab initio* dipole moment *Zemke* of Ref.[36].

Table S6: Reproduction of the reference electric dipole matrix elements $<vJ|\mu|v'J'>$ of the $X^1\Sigma^+$ state of HF by the empirical[29,32,34] and *ab initio*[30,31,35,36,42,50] electric dipole moment functions[a] .

N	v	J	v'	J'	$<vJ\|\mu_{exp}\|v'J'>^b$	weight	Δ^{29}	Δ^{34}	Δ^{32}	Δ^{31}	Δ^{35}	Δ^{36}	Δ^{42}	Δ^{50}
1	0	0	0	0	1.8265670[61]	0.300D+01	0.004	-0.000	0.001	0.006	0.015	0.002	0.002	0.006
2	1	0	1	0	1.8736930[61]	0.140D+01	0.005	-0.000	0.003	0.006	0.016	0.002	0.002	0.007
3	2	0	2	0	1.9232000[61]	0.000D+00	0.007	0.002	0.007	0.008	0.019	0.003	0.004	0.010
4	0	0	0	0	1.8265260[62]	0.300D+01	0.004	-0.000	0.001	0.006	0.015	0.002	0.002	0.006
5	1	0	1	0	1.8720000[63]	0.000D+00	0.004	-0.001	0.002	0.005	0.015	0.001	0.001	0.006
6	2	0	2	0	1.9185000[64]	0.000D+00	0.005	-0.001	0.004	0.005	0.016	0.001	0.002	0.007
7	3	0	3	0	1.9614000[64]	0.260D+00	0.005	-0.001	0.004	0.005	0.016	-0.000	0.001	0.007
8	0	0	0	0	1.8190000[29]	0.000D+00	0.000	-0.004	-0.003	0.001	0.011	-0.002	-0.002	0.002
9	1	0	1	0	1.8650000[29]	0.000D+00	0.000	-0.005	-0.001	0.001	0.011	-0.003	-0.002	0.002
10	2	0	2	0	1.9090000[29]	0.000D+00	-0.000	-0.006	-0.001	0.000	0.011	-0.004	-0.003	0.002
11	3	0	3	0	1.9530000[29]	0.000D+00	0.000	-0.005	0.000	0.000	0.012	-0.005	-0.003	0.003
12	4	0	4	0	1.9940000[29]	0.000D+00	0.000	-0.005	0.000	0.000	0.012	-0.005	-0.004	0.003
13	5	0	5	0	2.0320000[29]	0.000D+00	0.000	-0.003	-0.000	0.001	0.012	-0.005	-0.004	0.004
14	6	0	6	0	2.0660000[29]	0.000D+00	0.000	-0.002	-0.002	0.001	0.012	-0.004	-0.004	0.004
15	0	0	1	0	-0.0985000[29]	0.100D+02	-0.001	-0.014	0.075	-0.009	0.035	-0.036	-0.018	0.019
16	0	0	2	0	-0.0127000[29]	0.620D+03	0.000	0.031	0.138	0.014	0.055	-0.029	0.006	0.054
17	0	0	3	0	-0.0018700[29]	0.290D+05	0.004	0.195	0.160	0.062	0.003	-0.025	0.047	0.154
18	0	0	4	0	-0.0003600[29]	0.770D+06	-0.007	0.082	0.068	0.016	-0.205	-0.121	0.024	0.187
19	0	0	5	0	-0.0000816[29]	0.150D+08	-0.019	-0.262	-0.113	-0.064	-0.446	-0.171	-0.049	0.138
20	0	0	6	0	-0.0000218[29]	0.210D+09	-0.018	-0.533	-0.173	-0.085	-0.609	0.091	-0.100	0.087
21	1	0	2	0	-0.1380000[29]	0.530D+01	0.002	-0.012	0.072	-0.007	0.034	-0.034	-0.017	0.020
22	1	0	3	0	-0.0228000[29]	0.190D+03	0.002	0.010	0.134	0.008	0.053	-0.032	0.001	0.045
23	1	0	4	0	-0.0038900[29]	0.660D+04	0.001	0.181	0.162	0.063	0.023	-0.009	0.045	0.141
24	1	0	5	0	-0.0008620[29]	0.130D+06	0.002	0.161	0.135	0.046	-0.137	-0.079	0.046	0.205
25	1	0	6	0	-0.0002200[29]	0.210D+07	0.003	-0.148	-0.043	-0.026	-0.370	-0.150	-0.005	0.184
26	1	0	7	0	-0.0000633[29]	0.250D+08	-0.006	-0.451	-0.156	-0.094	-0.554	-0.049	-0.070	0.121
27	2	0	3	0	-0.1660000[29]	0.360D+01	0.001	-0.011	0.065	-0.008	0.030	-0.034	-0.018	0.020
28	2	0	4	0	-0.0334000[29]	0.900D+02	0.002	-0.011	0.130	-0.001	0.049	-0.038	-0.005	0.034
29	2	0	5	0	-0.0064000[29]	0.240D+04	0.001	0.158	0.153	0.063	0.037	0.004	0.042	0.126
30	2	0	6	0	-0.0015800[29]	0.400D+05	0.000	0.215	0.188	0.062	-0.081	-0.043	0.056	0.207
31	2	0	7	0	-0.0004440[29]	0.510D+06	0.005	-0.044	0.021	-0.008	-0.300	-0.131	0.017	0.205
32	2	0	8	0	-0.0001390[29]	0.520D+07	0.004	-0.355	-0.120	-0.095	-0.492	-0.109	-0.041	0.156
33	3	0	4	0	-0.1870000[29]	0.290D+01	0.000	-0.006	0.058	-0.007	0.027	-0.033	-0.019	0.020
34	3	0	5	0	-0.0447000[29]	0.500D+02	0.002	-0.030	0.129	-0.009	0.045	-0.046	-0.011	0.025
35	3	0	6	0	-0.0094200[29]	0.110D+04	0.003	0.126	0.132	0.059	0.044	0.009	0.037	0.110
36	3	0	7	0	-0.0025700[29]	0.150D+05	0.006	0.259	0.243	0.084	-0.024	0.002	0.070	0.213
37	3	0	8	0	-0.0007800[29]	0.160D+06	0.003	0.059	0.090	0.013	-0.229	-0.101	0.036	0.220
38	3	0	9	0	-0.0002590[29]	0.150D+07	-0.010	-0.262	-0.086	-0.083	-0.434	-0.145	-0.033	0.164
39	4	0	5	0	-0.2020000[29]	0.250D+01	-0.001	-0.001	0.047	-0.006	0.024	-0.030	-0.020	0.021
40	4	0	6	0	-0.0569000[29]	0.310D+02	0.002	-0.045	0.136	-0.017	0.043	-0.053	-0.016	0.018
41	4	0	7	0	-0.0129000[29]	0.600D+03	0.002	0.081	0.093	0.047	0.038	0.001	0.025	0.086
42	4	0	8	0	-0.0038300[29]	0.680D+04	0.003	0.270	0.273	0.095	0.016	0.035	0.073	0.204
43	4	0	9	0	-0.0012600[29]	0.630D+05	0.003	0.155	0.165	0.036	-0.158	-0.062	0.053	0.231
44	5	0	6	0	-0.2120000[29]	0.220D+01	0.000	0.016	0.035	0.001	0.023	-0.022	-0.016	0.027
45	5	0	7	0	-0.0701000[29]	0.200D+02	0.002	-0.055	0.154	-0.025	0.043	-0.060	-0.020	0.012
46	5	0	8	0	-0.0170000[29]	0.350D+03	0.007	0.039	0.051	0.035	0.031	-0.011	0.016	0.067
47	5	0	9	0	-0.0054300[29]	0.340D+04	0.003	0.263	0.288	0.109	0.048	0.065	0.075	0.192
48	6	0	7	0	-0.2150000[29]	0.220D+01	-0.001	0.031	0.007	0.006	0.016	-0.015	-0.016	0.029
49	6	0	8	0	-0.0848000[29]	0.140D+02	0.003	-0.057	0.193	-0.027	0.050	-0.060	-0.019	0.013
50	6	0	9	0	-0.0215000[29]	0.220D+03	0.004	-0.017	-0.010	0.007	0.007	-0.042	-0.006	0.035
51	7	0	8	0	-0.2110000[29]	0.220D+01	-0.002	0.051	-0.039	0.015	0.005	-0.005	-0.015	0.033
52	7	0	9	0	-0.1010000[29]	0.980D+01	0.001	-0.051	0.260	-0.027	0.062	-0.055	-0.017	0.016
53	8	0	9	0	-0.1980000[29]	0.260D+01	-0.000	0.074	-0.120	0.025	-0.016	0.005	-0.016	0.035
54	0	0	4	0	-0.0003480[65]	0.000D+00	-0.040	0.046	0.033	-0.018	-0.231	-0.151	-0.010	0.148
55	0	0	5	0	-0.0000879[65]	0.000D+00	0.056	-0.205	-0.045	0.008	-0.403	-0.107	0.026	0.226
56	0	9	1	8	-0.1229564[66]	0.660D+01	-0.001	-0.013	0.058	-0.007	0.030	-0.030	-0.016	0.015
57	0	8	1	7	-0.1208716[66]	0.680D+01	0.005	-0.007	0.066	-0.002	0.036	-0.025	-0.010	0.021
58	0	7	1	6	-0.1179986[66]	0.360D+02	0.005	-0.008	0.066	-0.002	0.036	-0.026	-0.011	0.021
59	0	6	1	5	-0.1153264[66]	0.230D+02	0.005	-0.007	0.069	-0.002	0.037	-0.026	-0.010	0.022
60	0	5	1	4	-0.1127909[66]	0.240D+02	0.007	-0.005	0.073	0.000	0.040	-0.025	-0.009	0.025
61	0	4	1	3	-0.1101271[66]	0.250D+02	0.008	-0.005	0.075	0.001	0.041	-0.025	-0.008	0.026
62	0	3	1	2	-0.1073631[66]	0.260D+02	0.007	-0.005	0.077	-0.000	0.041	-0.026	-0.009	0.026
63	0	2	1	1	-0.1047936[66]	0.270D+02	0.009	-0.004	0.080	0.001	0.043	-0.025	-0.008	0.028
64	0	1	1	0	-0.1022286[66]	0.290D+02	0.010	-0.003	0.084	0.002	0.045	-0.025	-0.007	0.029
65	0	0	1	1	-0.0971712[66]	0.530D+02	0.013	-0.000	0.092	0.005	0.050	-0.024	-0.005	0.033
66	0	1	1	2	-0.0945579[66]	0.340D+02	0.014	-0.000	0.095	0.005	0.051	-0.024	-0.005	0.035
67	0	2	1	3	-0.0917026[66]	0.360D+02	0.011	-0.003	0.095	0.002	0.049	-0.027	-0.008	0.033
68	0	3	1	4	-0.0890786[66]	0.380D+02	0.011	-0.003	0.098	0.002	0.050	-0.028	-0.008	0.033
69	0	4	1	5	-0.0865880[66]	0.400D+02	0.013	-0.002	0.102	0.003	0.053	-0.028	-0.007	0.036
70	0	5	1	6	-0.0843695[66]	0.420D+02	0.018	0.002	0.110	0.008	0.059	-0.024	-0.003	0.041
71	0	6	1	7	-0.0818482[66]	0.450D+02	0.019	0.003	0.115	0.009	0.061	-0.024	-0.002	0.043
72	0	7	1	8	-0.0788880[66]	0.640D+02	0.015	-0.002	0.114	0.004	0.058	-0.030	-0.007	0.039
73	0	8	1	9	-0.0764645[66]	0.510D+02	0.018	0.000	0.120	0.006	0.061	-0.029	-0.005	0.042
74	0	8	2	7	-0.0132212[67]	0.570D+03	-0.052	-0.026	0.058	-0.040	-0.005	-0.075	-0.048	-0.008
75	0	7	2	6	-0.0134239[67]	0.550D+03	-0.026	0.001	0.089	-0.013	0.023	-0.050	-0.020	0.020
76	0	6	2	5	-0.0131795[67]	0.580D+03	-0.032	-0.005	0.085	-0.020	0.016	-0.057	-0.027	0.014
77	0	5	2	4	-0.0128530[67]	0.610D+03	-0.045	-0.017	0.073	-0.033	0.003	-0.070	-0.041	0.002
78	0	4	2	3	-0.0131453[67]	0.580D+03	-0.012	0.017	0.112	0.001	0.039	-0.038	-0.006	0.038
79	0	3	2	2	-0.0129035[67]	0.600D+03	-0.019	0.011	0.107	-0.006	0.032	-0.046	-0.013	0.031
80	0	2	2	1	-0.0126925[67]	0.620D+03	-0.023	0.006	0.105	-0.010	0.028	-0.051	-0.018	0.027
81	0	1	2	0	-0.0126886[67]	0.620D+03	-0.012	0.018	0.121	0.001	0.040	-0.041	-0.007	0.040
82	0	0	2	1	-0.0123572[67]	0.650D+03	-0.015	0.016	0.124	-0.002	0.039	-0.045	-0.010	0.039
83	0	1	2	2	-0.0123410[67]	0.660D+03	-0.005	0.026	0.139	0.008	0.051	-0.036	0.000	0.050
84	0	2	2	3	-0.0120996[67]	0.680D+03	-0.013	0.018	0.133	0.000	0.044	-0.045	-0.008	0.043
85	0	3	2	4	-0.0119875[67]	0.700D+03	-0.010	0.021	0.140	0.003	0.048	-0.043	-0.006	0.046
86	0	4	2	5	-0.0117941[67]	0.720D+03	-0.015	0.016	0.139	-0.002	0.044	-0.048	-0.011	0.042
87	0	6	2	7	-0.0114455[67]	0.760D+03	-0.021	0.009	0.140	-0.009	0.041	-0.056	-0.018	0.037
88	0	7	2	8	-0.0114237[67]	0.770D+03	-0.011	0.019	0.156	0.001	0.053	-0.048	-0.008	0.049
89	0	8	2	9	-0.0112960[67]	0.780D+03	-0.010	0.019	0.162	0.002	0.056	-0.048	-0.007	0.050
90	0	3	2	2	-0.0129563[68]	0.600D+03	-0.015	0.015	0.112	-0.002	0.036	-0.042	-0.009	0.036
91	0	6	2	5	-0.0136648[68]	0.540D+03	0.003	0.032	0.124	0.016	0.054	-0.022	0.009	0.052
92	0	4	3	3	-0.0017380[69]	0.000D+00	-0.094	0.074	0.029	-0.042	-0.096	-0.115	-0.055	0.034
93	0	3	3	2	-0.0016770[69]	0.000D+00	-0.119	0.044	0.004	-0.069	-0.121	-0.141	-0.086	0.007
94	0	2	3	1	-0.0016420[69]	0.000D+00	-0.131	0.031	-0.005	-0.081	-0.133	-0.154	-0.101	-0.005
95	0	1	3	0	-0.0016390[69]	0.000D+00	-0.126	0.038	0.005	-0.076	-0.128	-0.151	-0.095	0.002
96	0	0	3	1	-0.0016230[69]	0.000D+00	-0.122	0.046	0.019	-0.071	-0.122	-0.149	-0.090	0.011
97	0	1	3	2	-0.0016130[69]	0.000D+00	-0.122	0.050	0.025	-0.070	-0.120	-0.149	-0.088	0.015
98	0	2	3	3	-0.0015630[69]	0.000D+00	-0.143	0.027	0.006	-0.092	-0.139	-0.170	-0.115	-0.007
99	0	3	3	4	-0.0016150[69]	0.000D+00	-0.108	0.072	0.054	-0.054	-0.102	-0.137	-0.071	0.036
100	0	4	3	5	-0.0015280[69]	0.000D+00	-0.150	0.024	0.010	-0.098	-0.142	-0.179	-0.124	-0.011
101	0	5	3	6	-0.0015740[69]	0.000D+00	-0.118	0.066	0.055	-0.063	-0.108	-0.149	-0.083	0.029
102	0	6	3	7	-0.0015540[69]	0.000D+00	-0.123	0.064	0.056	-0.068	-0.110	-0.154	-0.088	0.026

[a] $\Delta = (<vJ|\mu_{ref}|v'J'> - <vJ|\mu_{calc}|v'J'>)/<vJ|\mu_{ref}|v'J'>$. [b] The source reference of $<vJ|\mu_{ref}|v'J'>$ is given in parentheses.

Table S7: Parameters of the morphed electric dipole moment functions **ae-F12**-*spdfgh* of HF[42] obtained by fitting the reference dipole matrix elements given in Table S6. [a]

Parameter	Fit 1	Fit 2	Fit 3	Fit 4	Fit 5
			CCSD(T)		
r_e, Å	1.4999(48)	1.4767(39)	1.4718(55)	1.4941(49)	1.4917(28)
ρ_{ij}, Å	0.2549(17)	0.2468(15)	0.2531(18)	0.2467(22)	0.2363(93)
D_e, Debye	-2.4202(59)	-2.3884(41)	-2.3848(68)	-2.4085(62)	-2.4010(35)
α	1.0	1.337(41)	1.0	1.0	4.31(12)
β	1.0	1.0	0.824(22)	1.0	1.59[b]
δ	0.0	0.0	0.0	-0.00291(29)	0.0
σ^c_{fit}, Debye	0.0039	0.0027	0.0035	0.0039	0.0019
			CCSD		
r_e, Å	1.5608(72)	1.5200(32)	1.5142(55)	1.5528(64)	1.5195(32)
ρ_{ij}, Å	0.1735(24)	0.1598(12)	0.1700(24)	0.1848(16)	0.1604(11)
D_e, Debye	-2.4608(89)	-2.4152(40)	-2.4131(62)	-2.4599(80)	-2.4149(40)
α	1.0	3.44(15)	1.0	1.0	3.09(13)
β	1.0	1.0	0.607(23)	1.0	0.97[b]
δ	0.0	0.0	0.0	-0.53[b]	0.0
σ^c_{fit}, Debye	0.0054	0.0022	0.0028	0.0049	0.0021

[a]Fit 1,Fit 2,Fit 3,Fit 4 and Fit 5 performed using $[r_e, \rho_{ij}, D_e]$,$[r_e, \rho_{ij}, D_e, \alpha]$, $[r_e, \rho_{ij}, D_e, \beta]$,$[r_e, \rho_{ij}, D_e, \delta]$ and $[r_e, \rho_{ij}, D_e, \alpha, \beta]$, respectively. [b]Fixed after a preliminary determination. [c]The standard deviation of the fit.

Table S8: Reproduction of the reference electric dipole matrix elements $< v|\mu|v' >$ of the $X^1\Sigma^+$ state of DF by the reduced dipole moment functions constructed from their original literature empirical[29,34] and *ab initio*[30,35,42,50] variants[a].

N	v	v'	$<v\|\mu_{exp}\|v'>^b$	weight	Δ^{34}	Δ^{29}	Δ^{35}	Δ^{50}	Δ^{30}	Δ^{42}
1	0	0	1.8188050	0.300E+00	-0.0163950	0.0009020	-0.0082390	0.0001930	0.0004900	-0.0001350
2	0	0	1.8140000	0.000E+00	-0.0190870	-0.0017440	-0.0109090	-0.0024550	-0.0021580	-0.0027840
3	1	1	1.8480000	0.000E+00	-0.0162390	-0.0020220	-0.0077460	-0.0027210	-0.0024780	-0.0029630
4	2	2	1.8820000	0.000E+00	-0.0130560	-0.0020240	-0.0042200	-0.0027140	-0.0025270	-0.0028700
5	3	3	1.9150000	0.000E+00	-0.0099490	-0.0021480	-0.0007090	-0.0028210	-0.0026910	-0.0028920
6	4	4	1.9470000	0.000E+00	-0.0067650	-0.0022280	0.0029810	-0.0028620	-0.0027930	-0.0028470
7	5	5	1.9770000	0.000E+00	-0.0038540	-0.0025880	0.0065650	-0.0031450	-0.0031440	-0.0030410
8	6	6	2.0060000	0.000E+00	-0.0005300	-0.0025040	0.0107960	-0.0029230	-0.0030040	-0.0027270
9	7	7	2.0320000	0.000E+00	0.0023930	-0.0027340	0.0149430	-0.0029280	-0.0031090	-0.0026310
10	8	8	2.0560000	0.000E+00	0.0056290	-0.0024800	0.0197810	-0.0023320	-0.0026380	-0.0019210
11	9	9	2.0750000	0.000E+00	0.0079560	-0.0028580	0.0241930	-0.0022100	-0.0026820	-0.0016640
12	10	10	2.0900000	0.000E+00	0.0101280	-0.0029500	0.0290160	-0.0016000	-0.0022910	-0.0008870
13	11	11	2.1000000	0.000E+00	0.0119610	-0.0027100	0.0341560	-0.0003970	-0.0013800	0.0005330
14	12	12	2.1020000	0.000E+00	0.0123720	-0.0029060	0.0386520	0.0007070	-0.0006750	0.0019260
15	0	1	-0.0841000	0.140E+01	0.1160120	-0.0030470	0.1269950	-0.0019680	-0.0042800	0.0017710
16	0	2	-0.0083700	0.140E+03	0.0335900	0.0010750	0.0062480	0.0052640	0.0038870	0.0073540
17	0	3	-0.0008500	0.140E+05	0.0221270	0.0049930	-0.0302490	0.0085860	0.0076170	0.0096340
18	0	4	-0.0001260	0.630E+06	0.0279110	-0.0035670	0.1035980	-0.0314220	-0.0281680	-0.0314680
19	0	5	-0.0000255	0.150E+08	-0.0559590	-0.0220270	0.4422930	-0.0368560	-0.0352210	-0.0319550
20	0	6	-0.0000070	0.210E+09	-0.1318290	-0.0205150	-0.2574850	0.0676270	0.0613290	0.0810920
21	1	2	-0.1180000	0.720E+00	0.1186180	-0.0065770	0.1319230	-0.0057160	-0.0080940	-0.0018660
22	1	3	-0.0151000	0.440E+02	0.0344020	0.0006840	0.0115670	0.0043020	0.0029790	0.0064500
23	1	4	-0.0017700	0.320E+04	0.0128110	0.0007870	-0.0408520	0.0093940	0.0077620	0.0107880
24	1	5	-0.0002950	0.110E+06	0.0416720	0.0025300	-0.0196460	-0.0204910	-0.0174390	-0.0209390
25	1	6	-0.0000648	0.240E+07	-0.0208120	-0.0089710	0.1832460	-0.0324240	-0.0298920	-0.0311440
26	1	7	-0.0000189	0.280E+08	-0.0890030	0.0042780	0.0675250	0.0602960	0.0569040	0.0658470
27	2	3	-0.1440000	0.480E+00	0.1287630	-0.0021640	0.1441610	-0.0014120	-0.0038630	0.0025340
28	2	4	-0.0222000	0.200E+02	0.0341060	-0.0018050	0.0130070	0.0007420	-0.0004530	0.0029110
29	2	5	-0.0029300	0.120E+04	0.0066910	0.0012230	-0.0363820	0.0144020	0.0120750	0.0161020
30	2	6	-0.0005350	0.350E+05	0.0500300	0.0062610	-0.0702800	-0.0109810	-0.0081860	-0.0115630
31	2	7	-0.0001260	0.630E+06	0.0092290	0.0012540	0.0523260	-0.0280560	-0.0242450	-0.0266480
32	2	8	-0.0000373	0.720E+07	-0.0911830	-0.0147590	0.1513100	0.0158860	0.0145490	0.0207450
33	3	4	-0.1640000	0.370E+00	0.1342350	-0.0037840	0.1522250	-0.0029430	-0.0055230	0.0011440
34	3	5	-0.0299000	0.110E+02	0.0381140	-0.0010000	0.0165800	-0.0000340	-0.0010080	0.0021140
35	3	6	-0.0043300	0.530E+03	-0.0034020	-0.0011710	-0.0297330	0.0160500	0.0130100	0.0181740
36	3	7	-0.0008520	0.140E+05	0.0488620	0.0032690	-0.0921550	-0.0073240	-0.0050460	-0.0081320
37	3	8	-0.0002150	0.220E+06	0.0379750	0.0118820	-0.0324580	-0.0192000	-0.0150790	-0.0190130
38	3	9	-0.0000675	0.220E+07	-0.0356210	0.0165360	0.2129160	0.0250530	0.0253980	0.0289480
39	4	5	-0.1800000	0.310E+00	0.1404600	-0.0052140	0.1619680	-0.0039980	-0.0067700	0.0002640
40	4	6	-0.0381000	0.690E+01	0.0405690	-0.0032130	0.0162290	-0.0043050	-0.0049660	-0.0022300
41	4	7	-0.0060100	0.280E+03	-0.0113060	-0.0005510	-0.0190000	0.0199920	0.0162300	0.0225800
42	4	8	-0.0012600	0.630E+04	0.0450600	0.0008450	-0.0861380	-0.0025340	-0.0008800	-0.0033240
43	4	9	-0.0003310	0.910E+05	0.0420430	0.0000190	-0.1260010	-0.0308780	-0.0262880	-0.0309900
44	4	10	-0.0001080	0.860E+06	-0.0188110	0.0092140	0.1278760	0.0010300	0.0031360	0.0041310
45	5	6	-0.1930000	0.270E+00	0.1496560	-0.0037800	0.1761050	-0.0017870	-0.0048350	0.0026910
46	5	7	-0.0470000	0.450E+01	0.0462450	-0.0037020	0.0171510	-0.0073070	-0.0075450	-0.0053730
47	5	8	-0.0079600	0.160E+03	-0.0229590	-0.0031290	-0.0155200	0.0200010	0.0155220	0.0231460
48	5	9	-0.0017800	0.320E+04	0.0425260	0.0029150	-0.0573460	0.0070800	0.0079670	0.0063380
49	5	10	-0.0004910	0.410E+05	0.0594750	0.0051850	-0.1616860	-0.0223280	-0.0176820	-0.0231140
50	5	11	-0.0001630	0.380E+06	-0.0020410	0.0003350	-0.0129600	-0.0201210	-0.0167320	-0.0180700
51	6	7	-0.2020000	0.250E+00	0.1566670	-0.0054410	0.1901620	-0.0020990	-0.0055690	0.0026910
52	6	8	-0.0567000	0.310E+01	0.0552950	-0.0025070	0.0202360	-0.0090280	-0.0087230	-0.0073250
53	6	9	-0.0102000	0.960E+02	-0.0368900	-0.0077690	-0.0200920	0.0171040	0.0119200	0.0208680
54	6	10	-0.0024100	0.170E+04	0.0308550	-0.0015040	-0.0287440	0.0102710	0.0102590	0.0096990
55	6	11	-0.0006940	0.210E+05	0.0637710	0.0009650	-0.1643320	-0.0212130	-0.0167310	-0.0223910
56	6	12	-0.0002390	0.180E+06	0.0254170	0.0028270	-0.1447520	-0.0251990	-0.0207940	-0.0242410
57	7	8	-0.2080000	0.230E+00	0.1661600	-0.0045000	0.2092460	0.0009720	-0.0031110	0.0061860
58	7	9	-0.0671000	0.220E+01	0.0649530	-0.0027770	0.0233480	-0.0125790	-0.0116010	-0.0112070
59	7	10	-0.0129000	0.600E+02	-0.0404000	-0.0025260	-0.0206760	0.0228330	0.0170460	0.0272460
60	7	11	-0.0031800	0.990E+03	0.0174510	-0.0050230	-0.0036440	0.0141630	0.0131350	0.0138420
61	7	12	-0.0009550	0.110E+05	0.0652460	-0.0013050	-0.1283260	-0.0163040	-0.0122000	-0.0178150
62	8	9	-0.2100000	0.230E+00	0.1748400	-0.0045870	0.2309900	0.0041210	-0.0008740	0.0099600
63	8	10	-0.0783000	0.160E+01	0.0763960	-0.0035480	0.0281630	-0.0169320	-0.0151370	-0.0160180
64	8	11	-0.0159000	0.400E+02	-0.0520130	-0.0057150	-0.0341000	0.0192220	0.0128660	0.0243800
65	8	12	-0.0041300	0.590E+03	0.0062990	-0.0040500	0.0177860	0.0219560	0.0198180	0.0220490
66	9	10	-0.2080000	0.230E+00	0.1840140	-0.0036250	0.2579070	0.0098880	0.0035370	0.0166530
67	9	11	-0.0905000	0.120E+00	0.0913080	-0.0032880	0.0368350	-0.0204490	-0.0176830	-0.0201470
68	9	12	-0.0194000	0.270E+01	-0.0593110	-0.0057550	-0.0484850	0.0174770	0.0106760	0.0234000
69	10	11	-0.2010000	0.250E-01	0.1913290	-0.0035900	0.2897620	0.0170960	0.0086760	0.0252980
70	10	12	-0.1040000	0.920E-01	0.1114860	-0.0002620	0.0520830	-0.0212430	-0.0173480	-0.0217370
71	11	12	-0.1880000	0.280E-01	0.1943170	-0.0058260	0.3277140	0.0258350	0.0141380	0.0363700

[a] $\Delta = (<v|\mu_{ref}|v'> - <v|\mu_{calc}|v'>)/<v|\mu_{ref}|v'>$. [b] With the exception of $<0|\mu|0>$=1.818805 from Ref.,[62] the data are taken from Ref.[29]

Table S9: Reproduction of the reference electric dipole matrix elements $<v|\mu|v'>$ of the $X^1\Sigma^+$ state of HCl by the literature empirical,[34] *ab initio*[31] and 'dereduced' electric dipole moment functions[a].

N	v	J	v'	J'	$<vJ\|\mu_{ref}\|v'J'>$[b]	weight	Δ^{34}	Δ^{31}	Δ fit 1	Δfit 2	Δfit 3	Δfit 4	
1	0	0	0	0	1.1085700 [32]	0.000D+00	0.0003	0.0185	-0.0000	-0.0018	0.0011	-0.0027	-0.0013
2	0	0	0	0	1.1086000 [70]	0.810D+01	0.0003	0.0185	-0.0000	-0.0017	0.0011	-0.0027	-0.0013
3	1	0	1	0	1.1390000 [71]	0.770D+00	0.0003	0.0202	-0.0000	-0.0016	0.0008	-0.0019	-0.0010
4	2	0	2	0	1.1685000 [71]	0.730D+00	0.0005	0.0222	-0.0000	-0.0014	0.0005	-0.0009	-0.0005
5	3	0	3	0	1.1954652 [34]	0.000D+00	-0.0000	0.0233	-0.0010	-0.0021	-0.0009	-0.0005	-0.0008
6	4	0	4	0	1.2207652 [34]	0.000D+00	-0.0000	0.0249	-0.0018	-0.0026	-0.0021	0.0005	-0.0005
7	5	0	5	0	1.2428585 [34]	0.000D+00	-0.0000	0.0261	-0.0032	-0.0035	-0.0040	0.0015	-0.0005
8	6	0	6	0	1.2607525 [34]	0.000D+00	-0.0000	0.0269	-0.0053	-0.0051	-0.0066	0.0024	-0.0006
9	7	0	7	0	1.2733313 [34]	0.000D+00	0.0000	0.0272	-0.0082	-0.0074	-0.0103	0.0030	-0.0011
10	8	0	8	0	1.2793808 [34]	0.000D+00	0.0003	0.0267	-0.0122	-0.0105	-0.0152	0.0036	-0.0019
11	9	0	9	0	1.2776181 [34]	0.000D+00	0.0014	0.0255	-0.0172	-0.0146	-0.0213	0.0040	-0.0029
12	1	0	2	0	-0.0971000 [72]	0.000D+00	-0.0461	0.0147	-0.0385	-0.0394	-0.0522	-0.0299	-0.0408
13	2	0	3	0	-0.1187000 [72]	0.000D+00	-0.0268	0.0391	-0.0217	-0.0212	-0.0357	-0.0065	-0.0196
14	0	10	1	9	-0.0825672 [66]	0.150D+02	-0.0035	0.0520	0.0069	0.0051	-0.0044	0.0099	0.0022
15	0	9	1	8	-0.0816347 [66]	0.150D+02	-0.0024	0.0533	0.0081	0.0063	-0.0033	0.0111	0.0033
16	0	8	1	7	-0.0805362 [66]	0.150D+02	-0.0035	0.0525	0.0072	0.0054	-0.0044	0.0101	0.0023
17	0	7	1	6	-0.0796856 [66]	0.160D+02	-0.0017	0.0548	0.0093	0.0074	-0.0025	0.0121	0.0043
18	0	6	1	5	-0.0788346 [66]	0.160D+02	0.0002	0.0571	0.0113	0.0094	-0.0007	0.0141	0.0062
19	0	5	1	4	-0.0777839 [66]	0.170D+02	-0.0006	0.0566	0.0106	0.0088	-0.0014	0.0135	0.0054
20	0	4	1	3	-0.0767912 [66]	0.170D+02	-0.0008	0.0568	0.0107	0.0087	-0.0016	0.0135	0.0054
21	0	3	1	2	-0.0758356 [66]	0.170D+02	-0.0006	0.0575	0.0110	0.0091	-0.0014	0.0139	0.0057
22	0	2	1	1	-0.0748244 [66]	0.180D+02	-0.0012	0.0573	0.0106	0.0087	-0.0020	0.0135	0.0051
23	0	1	1	0	-0.0738562 [66]	0.180D+02	-0.0013	0.0577	0.0106	0.0087	-0.0022	0.0135	0.0051
24	0	0	1	1	-0.0719426 [66]	0.190D+02	-0.0016	0.0585	0.0107	0.0088	-0.0024	0.0138	0.0051
25	0	1	1	2	-0.0710403 [66]	0.200D+02	-0.0011	0.0597	0.0114	0.0094	-0.0020	0.0145	0.0057
26	0	2	1	3	-0.0702272 [66]	0.200D+02	0.0007	0.0622	0.0132	0.0113	-0.0003	0.0165	0.0075
27	0	3	1	4	-0.0694008 [66]	0.210D+02	0.0022	0.0645	0.0149	0.0129	0.0012	0.0183	0.0092
28	0	4	1	5	-0.0684209 [66]	0.210D+02	0.0014	0.0644	0.0143	0.0123	0.0004	0.0178	0.0085
29	0	5	1	6	-0.0676418 [66]	0.220D+02	0.0035	0.0674	0.0165	0.0146	0.0025	0.0202	0.0108
30	0	6	1	7	-0.0666449 [66]	0.230D+02	0.0024	0.0670	0.0155	0.0136	0.0013	0.0194	0.0098
31	0	7	1	8	-0.0656930 [66]	0.230D+02	0.0019	0.0673	0.0151	0.0133	0.0006	0.0193	0.0094
32	0	8	1	9	-0.0645739 [66]	0.240D+02	-0.0013	0.0648	0.0121	0.0103	-0.0026	0.0165	0.0064
33	0	9	1	10	-0.0639033 [66]	0.240D+02	0.0024	0.0697	0.0159	0.0141	0.0010	0.0206	0.0102
34	0	10	1	11	-0.0629444 [66]	0.250D+02	0.0016	0.0699	0.0152	0.0135	0.0001	0.0202	0.0096
35	0	8	2	7	-0.0084611 [73]	0.140D+03	0.0203	0.0121	0.0580	0.0433	0.0403	0.0054	0.0183
36	0	7	2	6	-0.0083235 [73]	0.140D+03	0.0088	0.0009	0.0475	0.0326	0.0293	-0.0056	0.0073
37	0	6	2	5	-0.0082541 [73]	0.150D+03	0.0052	-0.0025	0.0442	0.0292	0.0257	-0.0091	0.0038
38	0	4	2	3	-0.0080394 [73]	0.150D+03	-0.0125	-0.0197	0.0273	0.0121	0.0081	-0.0268	-0.0139
39	0	2	2	1	-0.0081930 [73]	0.150D+03	0.0136	0.0068	0.0528	0.0378	0.0335	-0.0000	0.0124
40	0	1	2	0	-0.0080978 [73]	0.150D+03	0.0049	-0.0015	0.0448	0.0297	0.0252	-0.0085	0.0039
41	0	0	2	1	-0.0078835 [73]	0.160D+03	-0.0167	-0.0222	0.0243	0.0088	0.0038	-0.0302	-0.0177
42	0	5	2	6	-0.0077537 [73]	0.170D+03	-0.0262	-0.0294	0.0161	0.0003	-0.0054	-0.0394	-0.0272
43	0	6	2	7	-0.0079875 [73]	0.160D+03	0.0036	0.0008	0.0456	0.0302	0.0246	-0.0085	0.0033
44	0	7	2	8	-0.0077384 [73]	0.170D+03	-0.0277	-0.0298	0.0152	-0.0008	-0.0066	-0.0408	-0.0287
45	0	8	2	9	-0.0079214 [73]	0.160D+03	-0.0050	-0.0065	0.0380	0.0223	0.0166	-0.0169	-0.0051
46	0	9	2	10	-0.0077240 [73]	0.170D+03	-0.0305	-0.0313	0.0131	-0.0030	-0.0089	-0.0434	-0.0314
47	0	10	2	11	-0.0079514 [73]	0.160D+03	-0.0029	-0.0030	0.0407	0.0250	0.0192	-0.0144	-0.0028
48	0	3	2	4	-0.0079927 [74]	0.160D+03	0.0021	-0.0023	0.0433	0.0280	0.0227	-0.0105	0.0016
49	0	3	2	4	-0.0080150 [75]	0.160D+03	0.0049	0.0005	0.0460	0.0307	0.0254	-0.0076	0.0044
50	0	4	2	3	-0.0080716 [75]	0.150D+03	-0.0086	-0.0157	0.0312	0.0160	0.0120	-0.0227	-0.0099
51	0	7	3	6	-0.0005757 [76]	0.300D+05	0.0471	-0.1060	0.0536	0.0385	-0.0268	0.0376	0.0430
52	0	5	3	4	-0.0005746 [76]	0.300D+05	0.0381	-0.1112	0.0479	0.0323	-0.0336	0.0302	0.0355
53	0	4	3	3	-0.0005625 [76]	0.320D+05	0.0123	-0.1321	0.0248	0.0087	-0.0589	0.0059	0.0113
54	0	3	3	2	-0.0005627 [76]	0.320D+05	0.0082	-0.1345	0.0220	0.0057	-0.0622	0.0022	0.0076
55	0	2	3	1	-0.0005552 [76]	0.320D+05	-0.0102	-0.1492	0.0050	-0.0119	-0.0811	-0.0160	-0.0106
56	0	1	3	0	-0.0005691 [76]	0.310D+05	0.0092	-0.1314	0.0252	0.0085	-0.0594	0.0038	0.0091
57	0	0	3	1	-0.0005531 [76]	0.330D+05	-0.0309	-0.1638	-0.0131	-0.0309	-0.1016	-0.0371	-0.0316
58	0	1	3	2	-0.0005672 [76]	0.310D+05	-0.0131	-0.1473	0.0061	-0.0115	-0.0808	-0.0183	-0.0128
59	0	2	3	3	-0.0005896 [76]	0.290D+05	0.0185	-0.1189	0.0378	0.0205	-0.0466	0.0134	0.0187
60	0	3	3	4	-0.0005683 [76]	0.310D+05	-0.0258	-0.1561	-0.0051	-0.0234	-0.0934	-0.0314	-0.0259
61	0	4	3	5	-0.0006242 [76]	0.260D+05	0.0612	-0.0796	0.0780	0.0611	-0.0032	0.0531	0.0582
62	0	5	3	6	-0.0006371 [76]	0.250D+05	0.0736	-0.0677	0.0893	0.0724	0.0090	0.0640	0.0690
63	0	7	3	8	-0.0006133 [76]	0.270D+05	0.0138	-0.1173	0.0369	0.0186	-0.0481	0.0085	0.0140
64	0	8	3	9	-0.0006404 [76]	0.240D+05	0.0477	-0.0866	0.0686	0.0508	-0.0137	0.0404	0.0457
65	0	3	3	2	-0.0005528 [77]	0.330D+05	-0.0096	-0.1498	0.0044	-0.0123	-0.0814	-0.0158	-0.0103
66	0	7	4	6	-0.0000288 [78]	0.000D+00	0.0396	-0.3284	0.2627	0.2469	-0.0532	0.2863	0.2468
67	0	6	4	5	-0.0000287 [78]	0.000D+00	0.0269	-0.3357	0.2555	0.2386	-0.0657	0.2754	0.2359
68	0	5	4	4	-0.0000286 [78]	0.000D+00	0.0168	-0.3413	0.2500	0.2318	-0.0758	0.2661	0.2268
69	0	4	4	3	-0.0000287 [78]	0.000D+00	0.0123	-0.3432	0.2483	0.2290	-0.0802	0.2606	0.2217

[a] $\Delta = (<vJ|\mu_{ref}|v'J'> - <vJ|\mu_{calc}|v'J'>)/<vJ|\mu_{ref}|v'J'>$. [b] The source reference of $<vJ|\mu_{ref}|v'J'>$ is given in parentheses.

Table S10: (Continuation of Table S9). Reproduction of the reference electric dipole matrix elements $<vJ|\mu|v'J'>$ of the $X^1\Sigma^+$ state of HCl by the literature empirical,[34] *ab initio*[31] and 'dereduced' electric dipole moment functions^a.

N	v	J	v'	J'	$<vJ\|\mu_{ref}\|v'J'>^b$	weight	Δ^{34}	Δ^{31}	Δfit 1	Δfit 2	Δfit 3	Δfit 4	Δfit 5
70	0	3	4	2	-0.0000291[78]	0.000D+00	0.0148	-0.3403	0.2517	0.2314	-0.0772	0.2602	0.2219
71	0	2	4	1	-0.0000296[78]	0.000D+00	0.0239	-0.3331	0.2597	0.2385	-0.0673	0.2645	0.2270
72	0	1	4	0	-0.0000305[78]	0.000D+00	0.0428	-0.3193	0.2741	0.2522	-0.0479	0.2754	0.2391
73	0	0	4	1	-0.0000316[78]	0.000D+00	0.0493	-0.3114	0.2800	0.2563	-0.0410	0.2749	0.2397
74	0	1	4	2	-0.0000316[78]	0.000D+00	0.0340	-0.3195	0.2696	0.2446	-0.0562	0.2614	0.2262
75	0	2	4	3	-0.0000320[78]	0.000D+00	0.0293	-0.3203	0.2664	0.2403	-0.0609	0.2552	0.2203
76	0	3	4	4	-0.0000324[78]	0.000D+00	0.0222	-0.3227	0.2610	0.2338	-0.0682	0.2470	0.2123
77	0	4	4	5	-0.0000328[78]	0.000D+00	0.0168	-0.3237	0.2566	0.2283	-0.0737	0.2398	0.2055
78	0	5	4	6	-0.0000335[78]	0.000D+00	0.0153	-0.3219	0.2547	0.2254	-0.0751	0.2354	0.2015
79	0	6	4	7	-0.0000341[78]	0.000D+00	0.0122	-0.3211	0.2512	0.2210	-0.0784	0.2295	0.1959
80	0	7	4	8	-0.0000347[78]	0.000D+00	0.0072	-0.3213	0.2460	0.2149	-0.0836	0.2220	0.1888
81	0	8	4	9	-0.0000354[78]	0.000D+00	0.0001	-0.3228	0.2388	0.2067	-0.0913	0.2126	0.1797
82	0	9	4	10	-0.0000361[78]	0.000D+00	-0.0070	-0.3242	0.2312	0.1981	-0.0991	0.2029	0.1703
83	0	7	5	6	0.0000081[78]	0.000D+00	-0.0083	0.5989	-0.2556	-0.2687	-0.0715	-0.2165	-0.2007
84	0	6	5	5	0.0000080[78]	0.000D+00	-0.0329	0.5576	-0.2854	-0.2979	-0.0940	-0.2442	-0.2279
85	0	5	5	4	0.0000080[78]	0.000D+00	-0.0502	0.5286	-0.3074	-0.3192	-0.1098	-0.2642	-0.2475
86	0	4	5	3	0.0000080[78]	0.000D+00	-0.0603	0.5118	-0.3207	-0.3316	-0.1179	-0.2757	-0.2586
87	0	3	5	2	0.0000081[78]	0.000D+00	-0.0617	0.5096	-0.3228	-0.3327	-0.1164	-0.2761	-0.2589
88	0	2	5	1	0.0000082[78]	0.000D+00	-0.0556	0.5201	-0.3150	-0.3237	-0.1065	-0.2670	-0.2496
89	0	1	5	0	0.0000084[78]	0.000D+00	-0.0404	0.5461	-0.2956	-0.3030	-0.0867	-0.2465	-0.2292
90	0	0	5	1	0.0000086[78]	0.000D+00	-0.0343	0.5612	-0.2924	-0.2972	-0.0767	-0.2395	-0.2217
91	0	1	5	2	0.0000086[78]	0.000D+00	-0.0455	0.5470	-0.3112	-0.3147	-0.0884	-0.2553	-0.2370
92	0	2	5	3	0.0000086[78]	0.000D+00	-0.0528	0.5401	-0.3256	-0.3278	-0.0963	-0.2668	-0.2480
93	0	3	5	4	0.0000087[78]	0.000D+00	-0.0523	0.5468	-0.3300	-0.3308	-0.0956	-0.2686	-0.2494
94	0	4	5	5	0.0000087[78]	0.000D+00	-0.0517	0.5550	-0.3351	-0.3342	-0.0951	-0.2708	-0.2511
95	0	5	5	6	0.0000088[78]	0.000D+00	-0.0474	0.5707	-0.3356	-0.3332	-0.0907	-0.2686	-0.2485
96	0	6	5	7	0.0000089[78]	0.000D+00	-0.0414	0.5909	-0.3346	-0.3305	-0.0847	-0.2647	-0.2442
97	0	7	5	8	0.0000090[78]	0.000D+00	-0.0350	0.6136	-0.3340	-0.3281	-0.0786	-0.2611	-0.2402
98	0	8	5	9	0.0000091[78]	0.000D+00	-0.0246	0.6452	-0.3288	-0.3211	-0.0685	-0.2532	-0.2317
99	0	9	5	10	0.0000092[78]	0.000D+00	-0.0124	0.6828	-0.3222	-0.3127	-0.0568	-0.2437	-0.2218
100	0	2	5	1	0.0000092[79]	0.000D+00	0.0557	0.6994	-0.1763	-0.1841	0.0102	-0.1333	-0.1178
101	0	1	5	0	0.0000089[79]	0.000D+00	0.0127	0.6316	-0.2277	-0.2347	-0.0297	-0.1812	-0.1647
102	0	0	5	1	0.0000092[79]	0.000D+00	0.0324	0.6691	-0.2089	-0.2134	-0.0072	-0.1594	-0.1428
103	0	1	5	2	0.0000096[79]	0.000D+00	0.0611	0.7198	-0.1794	-0.1827	0.0209	-0.1292	-0.1127
104	0	7	6	6	0.0000059[78]	0.290D+09	-0.0556	0.1278	-0.0510	-0.0772	-0.0114	-0.0571	-0.0578
105	0	6	6	5	0.0000060[78]	0.280D+09	-0.0581	0.1250	-0.0535	-0.0794	-0.0111	-0.0598	-0.0601
106	0	5	6	4	0.0000060[78]	0.270D+09	-0.0599	0.1231	-0.0555	-0.0811	-0.0104	-0.0620	-0.0617
107	0	4	6	3	0.0000061[78]	0.270D+09	-0.0597	0.1235	-0.0559	-0.0811	-0.0083	-0.0622	-0.0615
108	0	3	6	2	0.0000062[78]	0.260D+09	-0.0539	0.1305	-0.0505	-0.0750	-0.0008	-0.0564	-0.0553
109	0	2	6	1	0.0000064[78]	0.240D+09	-0.0429	0.1437	-0.0399	-0.0635	0.0115	-0.0450	-0.0436
110	0	1	6	0	0.0000066[78]	0.230D+09	-0.0240	0.1663	-0.0217	-0.0442	0.0308	-0.0259	-0.0242
111	0	0	6	1	0.0000068[78]	0.210D+09	-0.0153	0.1767	-0.0177	-0.0386	0.0381	-0.0196	-0.0175
112	0	1	6	2	0.0000069[78]	0.210D+09	-0.0266	0.1633	-0.0325	-0.0529	0.0255	-0.0330	-0.0308
113	0	2	6	3	0.0000069[78]	0.210D+09	-0.0321	0.1567	-0.0420	-0.0616	0.0180	-0.0409	-0.0385
114	0	3	6	4	0.0000070[78]	0.200D+09	-0.0336	0.1550	-0.0475	-0.0662	0.0142	-0.0445	-0.0421
115	0	4	6	5	0.0000071[78]	0.200D+09	-0.0324	0.1565	-0.0504	-0.0682	0.0126	-0.0455	-0.0430
116	0	5	6	6	0.0000072[78]	0.190D+09	-0.0290	0.1606	-0.0513	-0.0680	0.0130	-0.0442	-0.0418
117	0	6	6	7	0.0000074[78]	0.180D+09	-0.0243	0.1664	-0.0510	-0.0666	0.0144	-0.0418	-0.0394
118	0	7	6	8	0.0000075[78]	0.180D+09	-0.0182	0.1739	-0.0496	-0.0640	0.0169	-0.0380	-0.0357
119	0	8	6	9	0.0000077[78]	0.170D+09	-0.0117	0.1819	-0.0479	-0.0611	0.0195	-0.0340	-0.0317
120	0	9	6	10	0.0000078[78]	0.170D+09	-0.0083	0.1863	-0.0498	-0.0619	0.0189	-0.0335	-0.0312
121	0	2	6	1	0.0000070[79]	0.200D+09	0.0530	0.2582	0.0548	0.0333	0.1015	0.0501	0.0513
122	0	1	6	0	0.0000070[79]	0.200D+09	0.0368	0.2390	0.0382	0.0170	0.0876	0.0343	0.0358
123	0	0	6	1	0.0000072[79]	0.190D+09	0.0412	0.2442	0.0375	0.0177	0.0902	0.0357	0.0377
124	0	2	6	3	0.0000074[79]	0.180D+09	0.0336	0.2353	0.0243	0.0059	0.0805	0.0254	0.0275
125	0	6	7	5	0.0000028[78]	0.130D+10	-0.0983	0.0471	0.0634	0.0162	0.1131	-0.0114	-0.0031
126	0	5	7	4	0.0000029[78]	0.120D+10	-0.0823	0.0653	0.0796	0.0333	0.1321	0.0048	0.0141
127	0	4	7	3	0.0000030[78]	0.110D+10	-0.0582	0.0927	0.1021	0.0573	0.1564	0.0286	0.0386
128	0	3	7	2	0.0000032[78]	0.950D+09	-0.0001	0.1596	0.1527	0.1109	0.2063	0.0834	0.0935
129	0	2	7	1	0.0000031[78]	0.110D+10	-0.0678	0.0805	0.0886	0.0444	0.1483	0.0149	0.0262
130	0	1	7	0	0.0000032[78]	0.100D+10	-0.0568	0.0928	0.0959	0.0530	0.1565	0.0243	0.0357
131	0	0	7	1	0.0000033[78]	0.930D+09	-0.0504	0.0991	0.0934	0.0526	0.1553	0.0262	0.0375
132	0	1	7	2	0.0000034[78]	0.870D+09	-0.0367	0.1144	0.1010	0.0619	0.1623	0.0375	0.0483
133	0	2	7	3	0.0000035[78]	0.820D+09	-0.0250	0.1274	0.1061	0.0687	0.1664	0.0465	0.0567
134	0	5	7	6	0.0000036[78]	0.760D+09	-0.0446	0.1032	0.0672	0.0332	0.1261	0.0182	0.0264
135	0	6	7	7	0.0000038[78]	0.700D+09	-0.0266	0.1235	0.0769	0.0450	0.1334	0.0333	0.0404
136	0	7	7	8	0.0000038[78]	0.690D+09	-0.0365	0.1116	0.0596	0.0289	0.1151	0.0202	0.0264
137	0	8	7	9	0.0000038[78]	0.690D+09	-0.0548	0.0902	0.0331	0.0036	0.0880	-0.0019	0.0033

$^a\Delta = (<vJ|\mu_{ref}|v'J'> - <vJ|\mu_{calc}|v'J'>)/<vJ|\mu_{ref}|v'J'>$. ^{b} The source reference of $<vJ|\mu_{ref}|v'J'>$ is given in parentheses.

Table S11: Reproduction of the experimental electric dipole matrix elements $<vJ|\mu|v'J'>$ of the $X^1\Sigma^+$ state of $H^{79}Br$ by the *ab initio* electric dipole moment functions taken from the literature and by their reduced counterparts determined in this study[a].

N	v	J	v'	J'	$<vJ\|\mu_{ref}\|v'J'>$[b]	weight	Δ_f [34]	Δ_t [31]	Δ_f [31]	Δ_t [30]	Δ_f [30]
1	0	0	0	0	0.8265600 [80]	0.150D+02	-0.0020	-0.0003	-0.0044	-0.0012	-0.0046
2	1	0	1	0	0.8444990 [34]	0.000D+00	-0.0020	0.0021	-0.0032	0.0013	-0.0034
3	2	0	2	0	0.8619987 [34]	0.000D+00	-0.0020	0.0052	-0.0015	0.0043	-0.0017
4	3	0	3	0	0.8785173 [34]	0.000D+00	-0.0010	0.0084	0.0004	0.0075	0.0002
5	4	0	4	0	0.8936431 [34]	0.000D+00	-0.0010	0.0120	0.0026	0.0111	0.0023
6	5	0	5	0	0.9068563 [34]	0.000D+00	-0.0010	0.0159	0.0051	0.0149	0.0049
7	6	0	6	0	0.9174950 [34]	0.000D+00	-0.0010	0.0200	0.0080	0.0191	0.0081
8	7	0	7	0	0.9247165 [34]	0.000D+00	-0.0010	0.0242	0.0112	0.0239	0.0120
9	8	0	8	0	0.9274607 [34]	0.000D+00	-0.0010	0.0283	0.0146	0.0296	0.0174
10	0	0	1	1	-0.0383607 [81]	0.680D+02	-0.0030	0.0684	0.0491	0.0690	0.0478
11	0	1	1	2	-0.0384071 [81]	0.680D+02	0.0140	0.0851	0.0659	0.0857	0.0646
12	0	2	1	3	-0.0375378 [81]	0.710D+02	0.0070	0.0798	0.0601	0.0804	0.0588
13	0	3	1	4	-0.0370752 [81]	0.730D+02	0.0110	0.0843	0.0643	0.0850	0.0630
14	0	4	1	5	-0.0361711 [81]	0.760D+02	0.0020	0.0777	0.0571	0.0784	0.0558
15	0	6	1	7	-0.0353994 [81]	0.800D+02	0.0130	0.0906	0.0693	0.0913	0.0679
16	0	7	1	8	-0.0350529 [81]	0.810D+02	0.0190	0.0981	0.0764	0.0988	0.0750
17	0	0	1	1	-0.0383292 [81]	0.680D+02	-0.0040	0.0676	0.0483	0.0682	0.0471
18	0	1	1	2	-0.0382647 [81]	0.680D+02	0.0100	0.0817	0.0624	0.0823	0.0611
19	0	2	1	3	-0.0376599 [81]	0.710D+02	0.0100	0.0828	0.0632	0.0834	0.0619
20	0	3	1	4	-0.0369365 [81]	0.730D+02	0.0070	0.0809	0.0608	0.0815	0.0595
21	0	4	1	5	-0.0364438 [81]	0.750D+02	0.0090	0.0846	0.0642	0.0853	0.0628
22	0	6	1	7	-0.0357250 [81]	0.780D+02	0.0220	0.0988	0.0778	0.0996	0.0764
23	0	7	1	8	-0.0346793 [81]	0.830D+02	0.0080	0.0884	0.0665	0.0891	0.0651
24	0	2	2	1	-0.0029440 [82]	0.120D+05	0.0250	-0.1339	0.0236	-0.1312	0.0241
25	0	7	2	8	-0.0027680 [82]	0.130D+05	0.0350	-0.1243	0.0397	-0.1210	0.0401
26	0	8	2	7	-0.0030332 [83]	0.110D+05	-0.0180	-0.1777	-0.0244	-0.1750	-0.0238
27	0	7	2	6	-0.0030199 [83]	0.110D+05	-0.0090	-0.1681	-0.0142	-0.1655	-0.0137
28	0	6	2	5	-0.0029052 [83]	0.120D+05	-0.0340	-0.1998	-0.0398	-0.1970	-0.0392
29	0	5	2	4	-0.0028390 [83]	0.120D+05	-0.0440	-0.2137	-0.0500	-0.2108	-0.0494
30	0	4	2	3	-0.0028390 [83]	0.120D+05	-0.0330	-0.2003	-0.0368	-0.1975	-0.0362
31	0	3	2	2	-0.0027749 [83]	0.130D+05	-0.0440	-0.2152	-0.0479	-0.2123	-0.0474
32	0	2	2	1	-0.0030000 [83]	0.000D+00	0.0450	-0.1127	0.0418	-0.1100	0.0423
33	0	1	2	0	-0.0026646 [83]	0.140D+05	-0.0630	-0.2409	-0.0671	-0.2379	-0.0666
34	0	0	2	1	-0.0027240 [83]	0.130D+05	-0.0240	-0.1928	-0.0233	-0.1898	-0.0228
35	0	1	2	2	-0.0026981 [83]	0.140D+05	-0.0250	-0.1948	-0.0239	-0.1917	-0.0234
36	0	2	2	3	-0.0025884 [83]	0.150D+05	-0.0580	-0.2363	-0.0585	-0.2330	-0.0580
37	0	3	2	4	-0.0026038 [83]	0.150D+05	-0.0460	-0.2207	-0.0443	-0.2174	-0.0439
38	0	6	2	7	-0.0024166 [83]	0.000D+00	-0.1000	-0.2934	-0.1049	-0.2897	-0.1045
39	0	7	2	8	-0.0024940 [83]	0.000D+00	-0.0670	-0.2479	-0.0658	-0.2441	-0.0654
40	0	8	2	7	-0.0032241 [84]	0.960D+04	0.0440	-0.1079	0.0363	-0.1054	0.0368
41	0	7	2	6	-0.0031510 [84]	0.100D+05	0.0340	-0.1196	0.0280	-0.1170	0.0285
42	0	6	2	5	-0.0030469 [84]	0.110D+05	0.0130	-0.1440	0.0086	-0.1414	0.0091
43	0	5	2	4	-0.0029854 [84]	0.110D+05	0.0050	-0.1541	0.0015	-0.1515	0.0020
44	0	4	2	3	-0.0030031 [84]	0.110D+05	0.0230	-0.1347	0.0199	-0.1321	0.0204
45	0	3	2	2	-0.0029692 [84]	0.110D+05	0.0230	-0.1356	0.0207	-0.1329	0.0212
46	0	2	2	1	-0.0030017 [84]	0.110D+05	0.0450	-0.1121	0.0423	-0.1094	0.0429
47	0	0	2	0	-0.0028878 [84]	0.120D+05	0.0260	-0.1347	0.0254	-0.1319	0.0259
48	0	0	2	1	-0.0028283 [84]	0.130D+05	0.0140	-0.1488	0.0145	-0.1459	0.0150
49	0	1	2	2	-0.0027344 [84]	0.130D+05	-0.0120	-0.1789	-0.0103	-0.1759	-0.0098
50	0	2	2	3	-0.0028010 [84]	0.130D+05	0.0200	-0.1425	0.0218	-0.1394	0.0223
51	0	3	2	4	-0.0027423 [84]	0.130D+05	0.0050	-0.1590	0.0084	-0.1559	0.0089
52	0	4	2	5	-0.0027641 [84]	0.130D+05	0.0190	-0.1428	0.0229	-0.1396	0.0233
53	0	5	2	6	-0.0026896 [84]	0.140D+05	-0.0030	-0.1680	0.0019	-0.1646	0.0023
54	0	7	2	8	-0.0025452 [83]	0.150D+05	-0.0480	-0.2228	-0.0443	-0.2191	-0.0439
55	0	7	3	6	0.0003209 [83]	0.000D+00	-0.0490	0.3207	-0.1126	0.3308	-0.1207
56	0	6	3	5	0.0003406 [83]	0.860D+05	-0.0020	0.3478	-0.0613	0.3571	-0.0690
57	0	5	3	4	0.0003391 [83]	0.870D+05	-0.0170	0.3334	-0.0785	0.3425	-0.0861
58	0	4	3	3	0.0003421 [83]	0.850D+05	-0.0180	0.3282	-0.0811	0.3370	-0.0887
59	0	2	3	1	0.0003479 [83]	0.830D+05	-0.0200	0.3196	-0.0851	0.3281	-0.0924
60	0	1	3	0	0.0003550 [83]	0.790D+05	-0.0090	0.3244	-0.0733	0.3327	-0.0805
61	0	1	3	2	0.0003578 [83]	0.780D+05	-0.0250	0.3072	-0.0915	0.3152	-0.0986
62	0	2	3	3	0.0003606 [83]	0.770D+05	-0.0250	0.3064	-0.0907	0.3143	-0.0978
63	0	3	3	4	0.0003688 [83]	0.740D+05	-0.0090	0.3165	-0.0733	0.3242	-0.0802
64	0	4	3	5	0.0003728 [83]	0.720D+05	-0.0050	0.3192	-0.0678	0.3268	-0.0747
65	0	5	3	6	0.0003701 [83]	0.730D+05	-0.0180	0.3103	-0.0812	0.3180	-0.0881
66	0	6	3	7	0.0003742 [83]	0.710D+05	-0.0130	0.3145	-0.0745	0.3221	-0.0813
67	0	7	3	8	0.0003795 [83]	0.690D+05	-0.0040	0.3216	-0.0636	0.3292	-0.0703
68	0	8	3	9	0.0003873 [83]	0.670D+05	0.0120	0.3336	-0.0456	0.3411	-0.0521
69	0	6	4	5	0.0002074 [83]	0.230D+06	0.2040	0.1762	0.0231	0.1687	0.0183

[a] $\Delta = (<vJ|\mu_{ref}|v'J'> - <vJ|\mu_{calc}|v'J'>)/<vJ|\mu_{ref}|v'J'>$. Δ_t and Δ_f obtained using the original electric dipole moment function and its reduced counterpart, respectively. [b]The source reference of $<vJ|\mu_{ref}|v'J'>$ is given in parentheses.

Table S12: (Continuation of Table S11). Reproduction of the experimental electric dipole matrix elements $<vJ|\mu|v'J'>$ of the $X^1\Sigma^+$ state of $H^{79}Br$ by the *ab initio* electric dipole moment functions taken from the literature and by their reduced counterparts determined in this study[a].

N	v	J	v'	J'	$<vJ\|\mu_{ref}\|v'J'>^b$	weight	Δ_t [30]	Δ_f [30]	Δ_t [31]	Δ_f [31]	
70	0	5	4	4	0.0002131 [83]	0.220D+06	0.2250	0.1911	0.0410	0.1838	0.0366
71	0	4	4	3	0.0002149 [83]	0.220D+06	0.2240	0.1910	0.0412	0.1839	0.0370
72	0	3	4	2	0.0002182 [83]	0.210D+06	0.2310	0.1960	0.0473	0.1891	0.0433
73	0	2	4	1	0.0002186 [83]	0.210D+06	0.2220	0.1907	0.0412	0.1839	0.0374
74	0	1	4	0	0.0002149 [83]	0.220D+06	0.1900	0.1699	0.0166	0.1630	0.0127
75	0	0	4	1	0.0002173 [83]	0.210D+06	0.1810	0.1650	0.0109	0.1582	0.0071
76	0	1	4	2	0.0002276 [83]	0.190D+06	0.2260	0.1965	0.0482	0.1900	0.0445
77	0	2	4	3	0.0002267 [83]	0.190D+06	0.2100	0.1870	0.0369	0.1804	0.0330
78	0	3	4	4	0.0002214 [83]	0.200D+06	0.1710	0.1609	0.0058	0.1541	0.0016
79	0	4	4	5	0.0002276 [83]	0.190D+06	0.1930	0.1777	0.0255	0.1710	0.0213
80	0	5	4	6	0.0002218 [83]	0.200D+06	0.1530	0.1500	-0.0075	0.1430	-0.0122
81	0	6	4	7	0.0002315 [83]	0.190D+06	0.1930	0.1798	0.0275	0.1730	0.0227
82	0	5	5	4	0.0000799 [83]	0.160D+07	0.1730	0.1201	0.0034	0.1208	0.0303
83	0	4	5	3	0.0000727 [83]	0.190D+07	0.0560	0.0229	-0.1065	0.0243	-0.0763
84	0	3	5	2	0.0000744 [83]	0.180D+07	0.0690	0.0365	-0.0910	0.0383	-0.0610
85	0	2	5	1	0.0000790 [83]	0.160D+07	0.1230	0.0831	-0.0382	0.0851	-0.0097
86	0	1	5	0	0.0000717 [83]	0.190D+07	0.0080	-0.0203	-0.1553	-0.0179	-0.1237
87	0	0	5	1	0.0000794 [83]	0.160D+07	0.0930	0.0602	-0.0642	0.0623	-0.0358
88	0	1	5	2	0.0000721 [83]	0.190D+07	-0.0170	-0.0444	-0.1829	-0.0424	-0.1518
89	0	2	5	3	0.0000798 [83]	0.160D+07	0.0760	0.0466	-0.0801	0.0481	-0.0524
90	0	3	5	4	0.0000760 [83]	0.170D+07	0.0150	-0.0095	-0.1440	-0.0086	-0.1155
91	0	4	5	5	0.0000766 [83]	0.170D+07	0.0120	-0.0120	-0.1471	-0.0118	-0.1195
92	0	8	6	7	0.0000316 [85]	0.000D+00	0.0150	0.0856	-0.0022	0.1637	0.1079
93	0	7	6	6	0.0000246 [85]	0.000D+00	-0.2080	-0.1848	-0.2977	-0.0832	-0.1558
94	0	6	6	5	0.0000204 [85]	0.000D+00	-0.3460	-0.4482	-0.5853	-0.3239	-0.4130
95	0	5	6	4	0.0000180 [85]	0.000D+00	-0.4220	-0.6527	-0.8085	-0.5110	-0.6133
96	0	4	6	3	0.0000187 [85]	0.000D+00	-0.4040	-0.6121	-0.7637	-0.4744	-0.5747
97	0	3	6	2	0.0000206 [85]	0.000D+00	-0.3450	-0.4745	-0.6130	-0.3493	-0.4416
98	0	2	6	1	0.0000197 [85]	0.000D+00	-0.3780	-0.5572	-0.7034	-0.4258	-0.5240
99	0	1	6	0	0.0000178 [85]	0.000D+00	-0.4430	-0.7435	-0.9075	-0.5977	-0.7084
100	0	0	6	1	0.0000147 [85]	0.000D+00	-0.5490	-1.1523	-1.3560	-0.9765	-1.1153
101	0	1	6	2	0.0000227 [85]	0.000D+00	-0.3140	-0.4145	-0.5491	-0.3006	-0.3926
102	0	2	6	3	0.0000204 [85]	0.000D+00	-0.3920	-0.5906	-0.7429	-0.4646	-0.5691
103	0	3	6	4	0.0000207 [85]	0.000D+00	-0.3920	-0.5847	-0.7375	-0.4614	-0.5664
104	0	4	6	5	0.0000174 [85]	0.000D+00	-0.4970	-0.9054	-1.0906	-0.7601	-0.8877
105	0	5	6	6	0.0000189 [85]	0.000D+00	-0.4610	-0.7645	-0.9376	-0.6329	-0.7523
106	0	6	6	7	0.0000214 [85]	0.000D+00	-0.4030	-0.5807	-0.7373	-0.4657	-0.5739
107	0	7	6	8	0.0000244 [85]	0.000D+00	-0.3310	-0.3992	-0.5395	-0.3002	-0.3971
108	0	8	7	7	0.0000071 [85]	0.000D+00	0.2820	-0.8324	-0.8798	-0.5010	-0.5696
109	0	7	7	6	0.0000062 [85]	0.000D+00	0.1410	-1.1134	-1.1692	-0.7368	-0.8169
110	0	6	7	5	0.0000057 [85]	0.000D+00	0.0670	-1.3050	-1.3673	-0.9001	-0.9886
111	0	5	7	4	0.0000066 [85]	0.000D+00	0.2420	-1.0027	-1.0582	-0.6558	-0.7337
112	0	4	7	3	0.0000066 [85]	0.000D+00	0.2350	-1.0264	-1.0841	-0.6800	-0.7598
113	0	3	7	2	0.0000053 [85]	0.000D+00	-0.0280	-1.5730	-1.6482	-1.1386	-1.2413
114	0	2	7	1	0.0000053 [85]	0.000D+00	-0.0280	-1.5560	-1.6329	-1.1296	-1.2329
115	0	1	7	0	0.0000066 [85]	0.000D+00	0.1790	-1.0830	-1.1473	-0.7393	-0.8246
116	0	0	7	1	0.0000060 [85]	0.000D+00	0.0100	-1.3444	-1.4208	-0.9652	-1.0638
117	0	1	7	2	0.0000065 [85]	0.000D+00	0.0600	-1.1810	-1.2540	-0.8313	-0.9242
118	0	2	7	3	0.0000062 [85]	0.000D+00	-0.0330	-1.3264	-1.4064	-0.9564	-1.0569
119	0	3	7	4	0.0000069 [85]	0.000D+00	0.0260	-1.1244	-1.1995	-0.7891	-0.8820
120	0	4	7	5	0.0000072 [85]	0.000D+00	0.0310	-1.0447	-1.1190	-0.7242	-0.8149
121	0	5	7	6	0.0000064 [85]	0.000D+00	-0.1330	-1.3426	-1.4302	-0.9778	-1.0831
122	0	6	7	7	0.0000068 [85]	0.000D+00	-0.1290	-1.2394	-1.3256	-0.8928	-0.9948
123	0	7	7	6	0.0000066 [85]	0.000D+00	0.2200	-0.9772	-1.0294	-0.6249	-0.6998
124	0	7	8	6	0.0000018 [85]	0.000D+00	-0.1960	-2.0240	-1.9578	-1.5867	-1.6725
125	0	6	8	5	0.0000021 [85]	0.000D+00	-0.0550	-1.5483	-1.5022	-1.1917	-1.2655
126	0	5	8	4	0.0000019 [85]	0.000D+00	-0.1830	-1.9179	-1.8751	-1.5213	-1.6076
127	0	4	8	3	0.0000017 [85]	0.000D+00	-0.2550	-2.1670	-2.1299	-1.7471	-1.8425
128	0	3	8	2	0.0000019 [85]	0.000D+00	-0.2050	-1.9328	-1.9059	-1.5519	-1.6420
129	0	2	8	1	0.0000020 [85]	0.000D+00	-0.1920	-1.8532	-1.8332	-1.4888	-1.5780
130	0	1	8	0	0.0000017 [85]	0.000D+00	-1.4240	-2.3023	-2.2849	-1.8857	-1.9909
131	0	0	8	1	0.0000022 [85]	0.000D+00	-0.1560	-1.6343	-1.6265	-1.3062	-1.3930
132	0	1	8	2	0.0000022 [85]	0.000D+00	-0.1640	-1.6286	-1.6223	-1.3014	-1.3893
133	0	2	8	3	0.0000020 [85]	0.000D+00	-0.2560	-1.9136	-1.9075	-1.5497	-1.6487
134	0	3	8	4	0.0000021 [85]	0.000D+00	-0.2480	-1.8459	-1.8397	-1.4880	-1.5861
135	0	4	8	5	0.0000021 [85]	0.000D+00	-0.2660	-1.8815	-1.8741	-1.5152	-1.6161
136	0	5	8	6	0.0000021 [85]	0.000D+00	-0.3160	-2.0493	-2.0395	-1.6563	-1.7645
137	0	6	8	7	0.0000017 [85]	0.000D+00	-0.4660	-2.8600	-2.8440	-2.3539	-2.4927
138	0	7	8	8	0.0000020 [85]	0.000D+00	-0.3900	-2.3317	-2.3142	-1.8860	-2.0073

[a] $\Delta = (<vJ|\mu_{ref}|v'J'> - <vJ|\mu_{calc}|v'J'>)/<vJ|\mu_{ref}|v'J'>$. Δ_t and Δ_f obtained using the original electric dipole moment function and its reduced counterpart, respectively. [b] The source reference of $<vJ|\mu_{ref}|v'J'>$ is given in parentheses.

References

(1) Jenč, F. The reduced potential curve method for diatomic-molecules and its applications. *Adv. At. Mol. Phys.* **19**, 265-307 (1983).

(2) Jenč, F.; Brandt, B.A.; Špirko, V.; Bludský, O. Estimation of the ground-state potentials of alkali-metal diatomic-molecules with the use of the multiparameter generalized reduced-potential-curve method. *Phys. Rev. A* **48**, 1319-1327 (1993).

(3) Jenč, F. The reduced potential curve (RPC) method and its applications. *Int. Rev. Phys. Chem.***15**, 467-563 (1996).

(4) Born, M.; Oppenheimer R. Quantum theory of molecules. *Ann. Phys.* **84**, 0457-0484 (1927).

(5) Buckingham, A.D. Permanent and Induced Molecular Moments and Long-Range Intermolecular Forces. *Adv. At. Mol. Phys.* **12**, 107-142 (1967).

(6) Brown, J.M.; Colburn, E.A.; Watson, J.K.G.; Wayne F.D. Effective Hamiltonian for diatomic-molecules - Ab initio calculations of parameters of HCl^+. *J. Mol. Spectrosc.* **74**, 294-318 (1979).

(7) Augustovičová, L.D.; Špirko, V. Morphing radial molecular property functions of hydroxyl. *J. Quant. Spectrosc. Radiat. Transf.* **254**, 10721 (2020).

(8) Hollebeek, T.; Ho, T.S.; Rabitz, H. Constructing multidimensional molecular potential energy surfaces from ab initio data. *Ann. Rev. Phys. Chem.* **50**, 537-570 (1999).

(9) Špirko, V.; Li, X.; Paldus, J. Potential energy curve of N_2 revisited. *Collect. Czech. Chem. Commun.* **76**, 327-341 (2011).

(10) Stine, J.R.; Noid, D.W. A semiclassical inversion procedure for the dipole-moment function for diatomic molecules. *J. Chem. Phys.* **78**, 3647-3651 (1983).

(11) Trischka, J.; Salwen, H. Dipole moment function of diatomic molecules. *J. Chem. Phys.* **31**, 218-225 (1959).

(12) Ferguson, A.F.; Parkinson, D. The hydroxyl bands in the nightglow. *Planet. Space Sci.* **11**, 149-159 (1963).

(14) Henderson, R.D.E.; Shayesteh, A.; Tao, J.; Haugen, C.C; Bernath, P.F.; Le Roy, R.J. Accurate Analytic Potential and Born-Oppenheimer Breakdown Functions for MgH and MgD from a Direct-Potential-Fit Data Analysis. *J. Phys. Chem. A* **117**, 13373-13387 (2013).

(15) Yurchenko, S.N.; Lodi, L.; Tennyson, J.; Stolyarov, A.V. Duo: A general program for calculating spectra of diatomic molecules. *Comp. Phys. Commun.* **202**, 262-275 (2016).

(16) Medvedev, E.S.; Meshkov, V.V.; Stolyarov, A.V.; Ushakov, V.G.; Gordon, I.E. Impact of the dipole-moment representation on the intensity of high overtones. *J. Mol. Spectrosc.* **330**, 36-42 (2016).

(17) Ushakov, V.G.; Meshkov, V.V.; Ermilov, A.Yu.; Stolyarov, A.V.; Gordon, I.E.; Medvedev, E.S. Long-range potentials and dipole moments of the CO electronic states converging to the ground dissociation limit. *Phys. Chem. Chem. Phys.* **22**, 12058-12067 (2020).

(18) Medvedev, E.S.; Ushakov, V.G. Effect of the analytical form of the dipole-moment function on the rotational intensity distributions in the high-overtone vibrational bands of carbon monoxide. *J. Quant. Spectrosc. Radiat. Transf.* **272**, 107803 (2021).

(19) Meshkov, V.V.; Ermilov, A.Yu.; Stolyarov, A.V.; Medvedev, E.S.; Ushakov, V.G.; Gordon, I.E. Semi-empirical dipole moment of carbon monoxide and line lists for all its isotopologues revisited. *J. Quant. Spectrosc. Radiat. Transf.* **280**, 108090 (2022).

(20) Medvedev, E.S.; Ushakov, V.G. Irregular semi-empirical dipole-moment function for carbon monoxide and line lists for all its isotopologues verified for extremely high overtone transitions. *J. Quant. Spectrosc. Radiat. Transf.* **288**, 108255 (2022).

(21) Araújo, J.P.; Ballester, M.Y. A comparative review of 50 analytical representation of potential energy interaction for diatomic systems: 100 years of history. *Int. J. Quant. Chem.* **121**, e26808 (2021).

(22) Janzen, A.R.; Aziz, R.A. An accurate potential energy curve for helium based on ab initio calculations. *J. Chem. Phys.* **107**, 914-919 (1997).

(23) Špirko, V.; Sauer, S.P.A.; Szalewicz, K. Relation between properties of long-range diatomic bound states. *Phys. Rev. A* **87**, 012510 (2013).

(24) P. Soldán, P.; Špirko, V. Tuning ab initio data to scattering length: The a(3)Sigma(+) state of KRb. *J. Chem. Phys.* **127**, 121101 (2007).

(25) Patkowski, K.; Špirko, V.; Szalewicz, K. On the Elusive Twelfth Vibrational State of Beryllium Dimer. *Science* **326**, 1382-1384 (2009).

(26) Mallada, B., Gallardo, A.; Lamanec, M.; de la Torre, B.; Špirko, V.; Hobza, P.; Jelinek, P. Real-space imaging of anisotropic charge of sigma-hole by means of Kelvin probe force microscopy. *Science* **374**, 863-867 (2021).

(27) Chan, N.; Lin, C.; Jacobs, T.; Carpick, R.W.; Egberts, P. Quantitative determination of the interaction potential between two surfaces using frequency-modulated atomic force microscopy. *Beilstein J. Nanotechnol.* **11**, 729-739 (2020).

(28) Loukhovitski, B.I.; Sharipov, A.S.; Starik, A.M. Influence of vibrations and rotations of diatomic molecules on their physical properties: I. Dipole moment and static dipole polarizability. *J. Phys. B: At. Mol. Opt. Phys.* **49**, 125102 (2016).

(29) Sileo, R.N.; Cool, T.A. Overtone emission-spectroscopy of HF and DF - vibrational matrix-elements and dipole-moment function. *J. Chem. Phys.* **65**, 117-133 (1976).

(30) Harrison, J.F. Dipole and quadrupole moment functions of the hydrogen halides HF, HCl, HBr, and HI: A Hirshfeld interpretation. *J. Chem. Phys.***128**, 114320 (2008).

(31) Harrison, J.F. Private communication.

(32) Ogilvie, J. F. The electric dipole moment function of HF. *J. Phys. B* **21**, 1663-1671 (1988).

(33) Coxon, J.A.; Hajigeorgiou, P.G. Improved direct potential fit analyses for the ground electronic states of the hydrogen halides: HF/DF/TF, HCl/DCl/TCl, HBr/DBr/TBr and HI/DI/TI. *J. Quant. Spectrosc. Radiat. Transf.* **151**, 133-154 (2015).

(34) Li, G.; Gordon, I.E.; Le Roy, R.J.; Hajigeorgiou, P.G.; Coxon, J.A.; Bernath, P.F.; Rothman, L.S. Reference spectroscopic data for hydrogen halides. Part I: Construction and validation of the ro-vibrational dipole moment functions. *J. Quant. Spectrosc. Radiat. Transf.* **121**, 78-90 (2013).

(35) Piecuch, P.; Špirko, V.; Paldus, J. Vibrational dependence of the dipole moment and radiative transition probabilities in the X-1 Sigma(+) state of HF: a linear-response coupled-cluster study. *Mol. Phys.* **94**, 55-64 (1998).

(36) Zemke, W.T.; Stwalley, W.C.; Langhoff, S.R.; Valderrama, G.L.; Berry, M.J. Radiative transition-probabilities for all vibrational levels in the X1-SIGMA+ state of HF. *J. Chem. Phys.* **95**, 7846-7853 (1991).

(37) Buldakov, M.A.; Cherepanov, V.N. The semiempirical dipole moment funtions of the molecules HX (X=F, Cl, Br, I, O), CO, and NO. *J. Phys. B: At. Mol. Opt. Phys.* **37**, 3973-3986 (2004).

(38) Buldakov, M.A.; Koryukina, E.V.; Cherepanov, V.N.; Kalugina, Yu.N. Theoretical investigation into dipole-moment functions of HF, HCl, and HBr molecules at small internuclear separations. *Russ. Phys. J.* **49**, 1230-1235 (2006).

(39) Buldakov, M.A.; Koryukina, E.V.; Cherepanov, V.N.; Kalugina, Yu.N. Regularities in the behaviour of dipole moment functions of diatomic molecules at very small internuclear separations. *Phys. Rev. A* **2008**,*78*, 032516.

(40) Halkier, A.; Klopper, W.; Helgaker, T.; Jørgensen, P. Basis-set convergence of the molecular electric dipole moment. *J. Chem. Phys.* **111**, 4424-4430 (1999).

(41) Kahn, K.; Kirtman, B.; Noga, J.; Ten-no, S. Anharmonic vibrational analysis of water with traditional and explicitly correlated coupled cluster methods. *J. Chem. Phys.* **133** 074106 (2010).

(42) Kahn, K.; Kirtman, B.; Hagen, A.; Noga, J. Communication: Convergence of anharmonic infrared intensities of hydrogen fluoride in traditional and explicitly correlated coupled cluster calculations. *J. Chem. Phys.* **135**, 131103 (2011).

(43) Hait, D.; Head-Gordon, M. How Accurate is Density Functional Theory at Predicting Dipole Moments? *J. Chem. Theory Comput.* **14**, 1969-1981 (2018).

(44) Chrayteh, A.; Blondel, A.; Loos, P.F.; Jacquemin, D. Mountaineering Strategy to Excited States: Highly Accurate Oscillator Strengths and Dipole Moments of Small Molecules. *J. Chem. Theory Comput.* **17**, 416-438 (2021).

(45) Lykhin, A. O.; Truhlar, D. G.; Gagliardi, L. Dipole Moment Calculations Using Multiconfiguration Pair-Density Functional Theory and Hybrid Multiconfiguration Pair-Density Functional Theory. *J. Chem. Theory Comput.* **2021**,*17*, 7586-7601.

(46) Watson, J.K.G. Inversion of diatomic vibration-rotation expectation values. *J. Mol. Spectrosc.* **74**, 319-321 (1979).

(47) Sauer, S.P.A.; Špirko, V. Effective potential energy curves of the ground electronic state of CH+. *J. Chem. Phys.* **138**, 024315 (2013).

(48) Augustovičová, L.A.; Špirko, V. Radial molecular property functions of CH in its ground electronic state. *J. Quant. Spectrosc. Radiat. Transf.* **272**, 107809 (2021).

(49) Bielska, K.; Kyuberis, A.; Reed, Z. D.; Li, G.; Cygan, A.; Ciuryło, R.; Adkins, E. M.; Lodi, L.; Zobov, N. F.; Ebert, V.; Lisak, D.; Hodges, J. T.; Tennyson, J.; Polyansky, O. L. Subpromile Measurements and Calculations of CO (3-0) Overtone Line Intensities. *Phys. Rev. Lett.* **129**, 043002 (2022).

(50) Maroulis, G. Electric multipole moment, dipole and quadrupole (hyper)polarizability derivatives for HF ($X^1\Sigma^+$. *J. Mol. Struct.: THEOCHEM* **633**, 177-197 (2003).

(51) Vasilchenko, S.S.; Lyulin, O.M.; Perevalov, V.I. High sensitivity absorption spectroscopy of hydrogen chloride near 770nm. *J. Quant. Spectrosc. Radiat. Transf.* **296**, 108460 (2023).

(52) Coxon, J. A.; Hajigeorgiou, P. G. The B $^1\Sigma^+$ and X $^1\Sigma^+$ Electronic States of Hydrogen Fluoride: A Direct Potential Fit Analysis. *J. Phys. Chem. A* **110**, 6261-6270 (2006).

(53) Cardoen, W.; Gdanitz, R. J. Accurately solving the electronic Schrodinger equation of atoms and molecules using explicitly correlated (r(12)-) multireference configuration interaction. VII. The hydrogen fluoride molecule. *J. Chem. Phys.* **123**, 024304 (2005).

(54) Coxon, J. A.; Hajigeorgiou, P. G. The Radial Hamiltonians for the $X^1\Sigma^+$ and B $^1\Sigma^+$ States of HCl. *J. Mol. Spectrosc.* **203**, 49-54 (2000).

(55) Coxon, J. A.; Hajigeorgiou, P. G. Isotopic Dependence of Born-Oppenheimer Breakdown Effects in Diatomic Hydrides: The X $^1\Sigma^+$ States of HI/DI and HBr/DBr. *J. Mol. Spectrosc.* **150**, 1-27 (1991).

(56) Maroulis, G. A systematic study of basis set, electron correlation, and geometry effects on the electric multipole moments, polarizability, and hyperpolarizability of HCl. *J. Chem. Phys.* **108**, 5432-5448 (1998).

(57) Maroulis, G. Electric quadrupole-moment and quadrupole polarizability of hydrgen bromide. *J. Phys. B* **26**, 2957-2964 (1993).

(58) Somogyi, W.; Yurchenko, S. N.; Yachmenev, A. Calculation of electric quadrupole linestrengths for diatomic molecules: Application to the H_2, CO, HF, and O_2 molecules. *J. Chem. Phys.* **155**, 214303 (2021).

(59) Piecuch, P.; Kondo, A. E.; Špirko, V.; Paldus, J. Molecular quadrupole moment functions of HF and N_2. I. *Ab initio* linear-response coupled-cluster results. *J. Chem. Phys.* **104**, 4699-4715 (1996).

(60) Kondo, A. E.; Piecuch, P.; Paldus, J. Orthogonally spin-adapted single-reference coupled-cluster formalism: Linear response calculation of higher-order static properties. *J. Chem. Phys.* **104**, 8566-8585 (1996).

(61) Bass, S. M.; DeLeon, R. L.; Muenter, J. S. Stark, Zeeman, and hyperfine properties of v=0, v=1, and the equilibrium configuration of hydrogen fluoride. *J. Chem. Phys.* **86**, 4305 (1987).

(62) Muenter, J. S.; Klemperer, W. Hyperfine Structure Constants of HF and DF. *J. Chem. Phys.* **52**, 6033-6037 (1970).

(63) Gough, T. E.; Miller, R. E.; Scoles, G. Sub-Doppler resolution infrared molecular-beam spectroscopy - Stark-effect measurement of the dipole-moment of hydrogen-fluoride and hydrogen-cyanide in excited vibrational-states. *Faraday Discuss. Chem. Soc.* **71**, 77 (1981).

(64) Barnes, J. A.; Gough, T. E.; Stoer, M. The vibrational dependence of the dipole-moment of hydrogen-fluoride. *Can. J. Chem.* **72**, 499 (1994).

(65) Rimpel, G. Linienstärken in der 4-0 und 5-0 Rotationsschwingungsbande von Fluorwasserstoff. *Z. Naturforsch.* **29a**, 588-592 (1974).

(66) Pine, A. S.; Fried, A.; Elkins, J. W. Spectral Intensities in the fundamental bands of HF and HCl. *J. Mol. Spectrosc.* **109**, 30 (1985).

(67) Meredith, R. E. Strengths and widths in the first overtone band of hydrogen fluoride. *J. Quant. Spectrosc. Radiat. Transfer* **12**, 485-503 (1972).

(68) Chou, S.-I.; Baer, D. S.; Hanson, R. K. Spectral intensity and lineshape measurements in the first overtone band of HF using tunable diode lasers. *J. Mol. Spectrosc.* **195**, 123-131 (1999).

(69) Spellicy, R. L.; Meredith, R. E.; Smith, F. G. Stregths and collision broadened widths in second overtone band of hydrogen-fluoride. *J. Chem. Phys.* **57**, 5119 (1972).

(70) De Leeuw, F. H.; Dymanus, A. Magnetic Properties and Molecular Quadrupole Moment of HF and HCl by Molecular-Beam Electric-Resonance Spectrosopy. *J. Mol. Spectrosc.* **48**, 427-445 (1973).

(71) Kaiser, W. W. Dipole Moment and Hyperfine Parameters of $H^{35}Cl$ and $D^{35}Cl$. *J. Chem. Phys.* **53**, 1686-1703 (1970).

(72) Smith, R. G. Dipole moment function and vibration-rotation matrix elements of HCl^{35} and DCl^{35}. *J. Quant. Spectrosc. Radiat. Transfer* **13**, 717-739 (1973).

(73) Toth, R. A.; Hunt, R. H.; Plyler, E. K. Line strengths, line widths, and dipolemoment function for HCl. *J. Mol. Spectrosc.* **35**, 110-126 (1970).

(74) Ortwein, P.; Woiwode, W.; Wagner, S.; Gisi, M.; Ebert, V. Laser-based measurements of line strength, self- and pressure-broadening coefficients of the $H^{35}Cl$ R(3) absorption line in the first overtone region for pressures up to 1 MPa. *Appl. Phys. B: Lasers Opt.* **100**, 341-347 (2010).

(75) De Rosa, M.; Nardini, C.; Piccolo, C.; Corsi, C.; D'Amato, F. Pressure broadening and shift of transitions of the first overtone of HCl. *Appl. Pys. B: Lasers Opt.* **72**, 245-248 (2001).

(76) Ogilvie, J. F.; Lee, Y.-P. Linestrengths in the 3-0 vibration-rotational band of gaseous $^1H^{35}Cl$ and electric dipole moment function. Chem. Phys. Letters **159**, 239-243 (1989).

(77) Stanton, A. C.; Silver, J. A. Measurements in the HCl 3-0 band using a near-IR InGaAsP diode laser. *Appl. Opt.* **27**, 5009-5015 (1988).

(78) Gelfand, J.; Zughull, M.; Rabitz, H.; Han, C. J. Absorption intensities for the 4-0 through 7-0 overtone bands of HCl. *J. Quant. Spectrosc. Radiat. Transfer* **26**, 303-305 (19

(79) Reddy, K. V. High-resolution measurtement of HCl overtone vibration-rotation bands by intracavity dye laser techniques. *J. Mol. Spectrosc.* **82**, 127-137 (1980).

(80) Dabbousi, O. B.; Meerts, W. L.; De Leeuw, F. H.; Dymanus, A. Stark-Zeeman hyperfine structure of $H^{79}Br$ and $H^{81}Br$ by molecular-beam electric-resonance spectroscopy. *Chem. Phys.* **2**, 473-477 (1973).

(81) Seoudi, B.; Henry, A.; Margottin-Maclou, M. Intensities, self-broadening and CO_2-broadening parameters in the fundamental band of HBr. *J. Mol. Spectrosc.* **112**, 88-94 (1985).

(82) Chou, S.-I.; Baer, D. S.; Hanson, R. K. High-resolution measurements of HBr transitions in the first overtone band using tunable diode lasers. *J. Mol. Spectrosc.* **200**, 138-142 (2000).

(83) Bernage, P.; Niay, P. Absorption intensities for vibration-rotation bands and the dipole-moment expansion of HBr. *J. Quant. Spectrosc. Radiat.Transfer* **18**, 315-325 (1977).

(84) Babrov, H. J.; Shabott, A. L.; Rao, B. S. Matrix Elements for Vibration-Rotation Transitions in HBr Overtone and Hot Bands. *J. Chem. Phys.* **42**, 4124-4131 (1965).

(85) Carlisle, C. B.; Riris, H.; Wang, L. G.; Janik, G. R.; Gallagher, T. F.; Lopez Piñeiro, A.; Tipping, R. H. Measurement of High Overtone Intensities of HBr by Two-Tone Frequency-Modulation Spectroscopy. *J. Mol. Spectrosc.* **130**, 395-406 (1988).
